# Synthesis, Herbicidal Activity, Mode of Action, and In Silico Analysis of Novel Pyrido[2,3-*d*]pyrimidine Compounds

**DOI:** 10.3390/molecules28217363

**Published:** 2023-10-31

**Authors:** Lijing Min, Wei Liang, Joanna Bajsa-Hirschel, Peng Ye, Qiao Wang, Xinpeng Sun, Charles L. Cantrell, Liang Han, Nabo Sun, Stephen O. Duke, Xinghai Liu

**Affiliations:** 1 Key Laboratory of Vector Biology and Pathogen Control of Zhejiang Province, College of Life Science, Huzhou University, Huzhou 313000, China; minlijing@zjhu.edu.cn; 2 College of Chemical Engineering, Zhejiang University of Technology, Hangzhou 310014, China; w.lianghcez@outlook.com (W.L.); w.qiao@cnhu.com (Q.W.); sdzpsxp98@163.com (X.S.); hanliang@zjut.edu.cn (L.H.); 3 Natural Products Utilization Research Unit, Agricultural Research Service, U.S. Department of Agriculture, University, MS 38677, USA; joanna.bajsa-hirsche@usda.gov (J.B.-H.); charles.cantrell@usda.gov (C.L.C.); 4 Shanghai Souguo Science & Technology Co. Ltd., Shanghai 201708, China; 18901710008@189.cn; 5 College of Biology and Environmental Engineering, Zhejiang Shuren University, Hangzhou 310015, China; 6 National Center for Natural Products Research, School of Pharmacy, University of Mississippi, University, MS 38677, USA

**Keywords:** natural product, uracil, pyrido[2,3-*d*] pyrimidine derivatives, one-pot synthesis, herbicidal activity, protoporphyrinogen oxidase

## Abstract

Natural products are a main source of new chemical entities for use in drug and pesticide discovery. In order to discover lead compounds with high herbicidal activity, a series of new pyrido[2,3-*d*] pyrimidine derivatives were designed and synthesized using 2-chloronicotinic acid as the starting material. Their structures were characterized with ^1^H NMR, ^13^C NMR and HRMS, and the herbicidal activities against dicotyledonous lettuce (*Lactuca sativa*), field mustard (*Brassica campestris*), monocotyledonous bentgrass (*Agrostis stolonifera*) and wheat (*Triticum aestivum*) were determined. The results indicated that most of the pyrido[2,3-*d*] pyrimidine derivatives had no marked inhibitory effect on lettuce at 1 mM. However, most of the pyrido[2,3-*d*] pyrimidine derivatives possessed good activity against bentgrass at 1 mM. Among them, the most active compound, 3-methyl-1-(2,3,4-trifluorophenyl)pyrido[2,3-*d*]pyrimidine-2,4(1*H*,3*H*)-dione (**2o**), was as active as the positive controls, the commercial herbicides clomazone and flumioxazin. Molecular simulation was performed with molecular docking and DFT calculations. The docking studies provided strong evidence that **2o** acts as an herbicide by inhibition of protoporphyrinogen oxidase. However, the physiological results indicate that it does not act on this target in vivo, implying that it could be metabolically converted to a compound with a different molecular target.

## 1. Introduction

**Figure 1:**
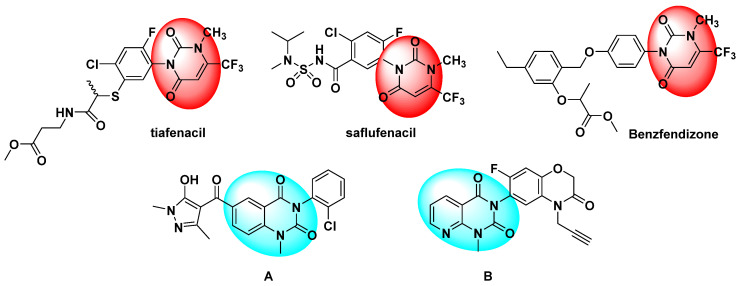
Bioactive molecules with a uracil skeleton.

Heterocyclic molecules are important scaffolds for synthetic and natural derivatives because of their wide range of biological activities [1,2,3,4,5]. Uracils are a class of natural bioactive compounds that exist in humans, animals, plants and microorganisms and possess diverse biological activities [6,7,8,9,10]. Hence, uracils are important nitrogen-linked heterocyclic molecules, which have received extensive attention in the field of agricultural chemistry because of their outstanding insecticidal, fungicidal and herbicidal bioactivities [11,12,13,14,15]. Among them, the uracil skeleton is a key active group in natural compounds and synthetic compounds. For example, the protoporphyrinogen oxidase (PPO) inhibitors tiafenacil [16] and benzfendizone (Figure 1) [17] have been successfully used to control weeds in cotton, corn and cereal crops. Another PPO inhibitor, saflufenacil (Figure 1) [18], controls broadleaf weeds and weeds that are resistant to glyphosate. These PPO inhibitors contain the uracil motif. The structure–activity relationships of these PPO inhibitors [19] show that the compounds have good herbicidal activity when the 1-position is an amino or methyl group, the 3-position is a substituted aryl group, and the 6-position is a trifluoromethyl group. Recently, research [20,21] reported that compounds A and B (Figure 1) were found to have significant herbicidal activity. Similarly, the pyridine ring is also an important active heterocycle, which is widely used in agricultural chemistry [22,23,24]. As a bioisostere of a benzene ring, it has a similar structure, but it is quite different from the benzene ring in some aspects, such as its oil–water partition coefficient properties [25]. Generally, when the benzene ring is replaced by a pyridine ring, it typically can improve the biological activity, such as with the commercial herbicides chlorpyridin, fluthiopyr and others. Some references reviewed the synthesis of a pyrido[2,3-*d*]pyrimidin-7(8*H*)-ones and biomedical applications [26,27,28,29].

**Figure 2:**
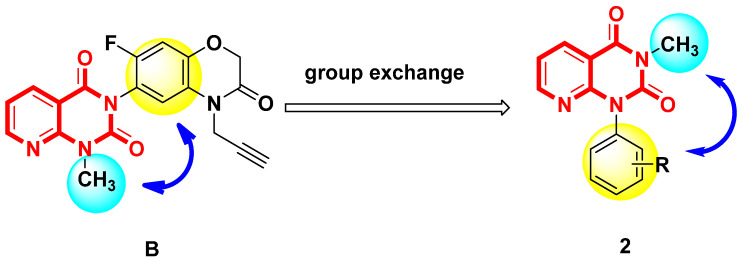
Design strategy of target compound **2**.

In our previous work, many pyridine or pyrimidine derivatives [30,31] were synthesized, some of which displayed good herbicidal activity. For example, compound B was reported by Wang et al. [19] to have good herbicidal activity. In the present study, the methyl group and benzene ring positions were exchanged (Figure 2). A series of pyrido[2,3-*d*]pyrimidine derivatives were designed and synthesized, and their herbicidal activity was studied.

## 2. Results and Discussion

### 2.1. Synthesis and Spectra Analysis

**Scheme 1:**
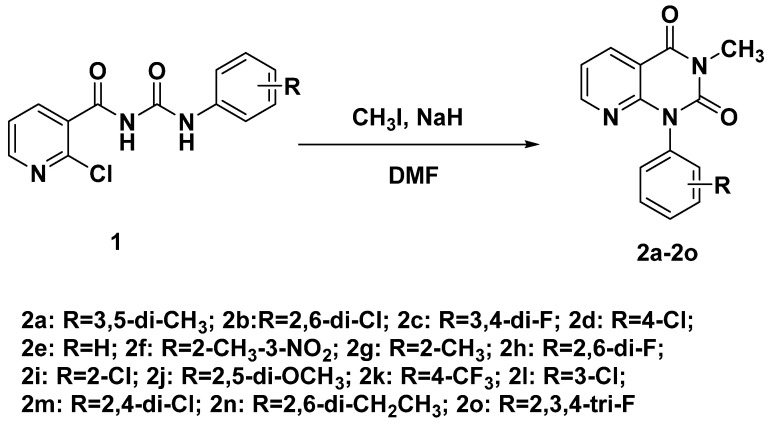
Synthetic route of pyrido[2,3-*d*]pyrimidine compound **2**.

The synthetic process of pyrido[2,3-*d*]pyrimidine compounds is shown in Scheme 1. The general synthesis method of pyrido[2,3-*d*]pyrimidine is 2-aminonicotinamide and phosgene as the starting materials. In this paper, 2-chloro-*N*-(phenylcarbamoyl)nicotinamide was used as the starting material. The key intermediate acyl urea compounds **1** were synthesized in our lab according to reported work [32]. The starting material, 2-chloronicotinic acid, underwent four steps to give the acyl urea compounds. Finally, the key intermediate acyl urea compounds were cyclized under the base NaH via an intramolecular reaction.

In this step, we found that the reaction is greatly affected by bases. At first, the reaction used K_2_CO_3_ as a base at room temperature, and it did not work. When NaOH was used as a base at room temperature, it was still unsuccessful. When the reaction is performed under reflux, the reaction system is more complicated, and the product is not easy to separate and purify. At last, we found that, when NaH was used as the base under reflux conditions, the desired products were produced with good yield and purity. On the other hand, the reaction temperature is also an important factor for this reaction. We carried out the experiments below (Table 1). From Table 1, when we used THF as the solvent, the yield increased, while the reaction temperature increased. When the reaction temperature is at 50 °C, the yield is 70%. Due to the low boiling point of THF, we also tried DMF as a solvent, and the reaction time was prolonged. The best reaction condition is at 50 °C for 8 h in DMF.

All the structures of pyrido[2,3-*d*]pyrimidine compound **2** were tested with proton nuclear magnetic resonance spectroscopy and high-resolution mass spectrometry. In the ^1^H NMR spectra of pyrido[2,3-*d*]pyrimidine compound **2**, the methyl proton signals of the pyrido[2,3-*d*]pyrimidine compounds **2a**~**2o** can be found at approximately 3.5 ppm as a single peak. The pyridine ring and benzene ring proton signals are at 6.5~8.5 ppm. Finally, all the ESI-HRMS results of pyrido[2,3-*d*]pyrimidine compound **2** are according to the theoretical values.

### 2.2. Crystal Structure

**Figure 3:**
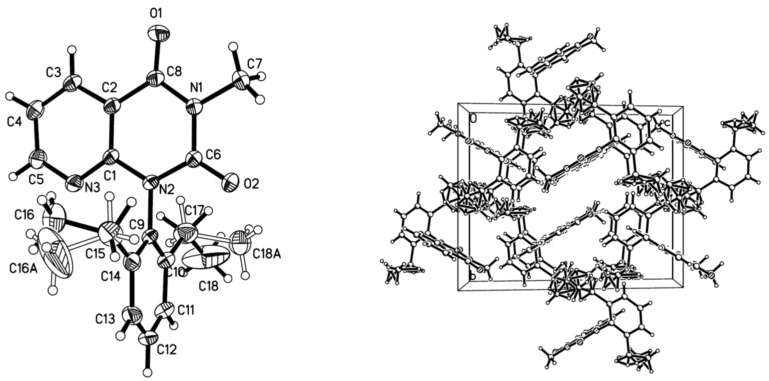
The crystal structure of compound **2n**.

The compound 1-(2,6-diethylphenyl)-3-methylpyrido[2,3-*d*]pyrimidine-2,4(1*H*,3*H*)-dione **2n** was diffracted with X-ray analysis, and the molecular structure is illustrated in Figure 3. From Figure 3, the benzene ring is nearly vertical with the pyrido[2,3-*d*]pyrimidine-2,4(1*H*,3*H*)-dione ring, the dihedral angle (*θ*) is 88.2° with the plane equation 7.236*x* + −2.922*y* + −10.192*z* = 1.7607 and −0.302*x* + 10.671*y* + −7.561*z* = 8.8098, respectively, and the largest deviation is 0.0069 and 0.0070 nm. Compound **2n** has intermolecular hydrogen bonds via C-H•••N and C-H•••O interactions in Figure 1. Between the pyrido[2,3-*d*]pyrimidine-2,4(1*H*,3*H*)-dione ring and the CH_3_ groups (one is on pyrido[2,3-*d*]pyrimidine-2,4(1*H*,3*H*)-dione, the other is an Et group), there are two intermolecular edge-to-face π–π stackings in the crystal with a centroid distance of 3.065 Å and 3.102 Å, respectively. The hydrogen bonds and edge-to-face π–π stackings interactions formed infinite three-dimensional net structures.

### 2.3. Phytotoxic Effects

As in many other papers, we routinely determine the herbicidal activities of new compounds on standard monocotyledonous (bentgrass) and dicotyledonous (lettuce) species (e.g., [33]) because some herbicides only affect one of these general categories of weeds. The herbicidal activity of the compounds **2a**–**2o** against lettuce and bentgrass at a concentration of 1 mM is listed in Table 2. From Table 2, most of the pyrido[2,3-*d*]pyrimidine derivatives exhibited no activity against dicotyledonous plant lettuce except for compounds **2c**~**2g** and **2m**~**2o,** which had weak activity (1–2 ranking). Most of the pyrido[2,3-*d*]pyrimidine compounds exhibited good activity (4–5 ranking) against the monocotyledon plant bentgrass at 1 mM. Among these compounds, **2o** possessed the best activity against bentgrass, which is the same as the commercial herbicide-positive controls. From the herbicidal activity data, the inhibition rate of bentgrass was significantly higher than that of lettuce. The general structure–activity relationship indicated better herbicidal activity against monocots (bentgrass) than that on dicots (lettuce). For the monocots, when the -OCH_3_ was introduced into the benzene ring, the herbicidal activity was significantly reduced, such as compound **2j**. When the halogen was introduced into the benzene ring, the activity was significantly improved, especially for the fluorine substitution.

All compounds had reduced growth of field mustard in darkness and wheat in light at 100 ppm (ca. 300 nM) (Table 3). Compound **2o** was the most active compound against wheat with almost as much activity as flumioxazin at 10 ppm (ca. 30 nM).

### 2.4. Molecular Docking with NtPPO

**Figure 4:**
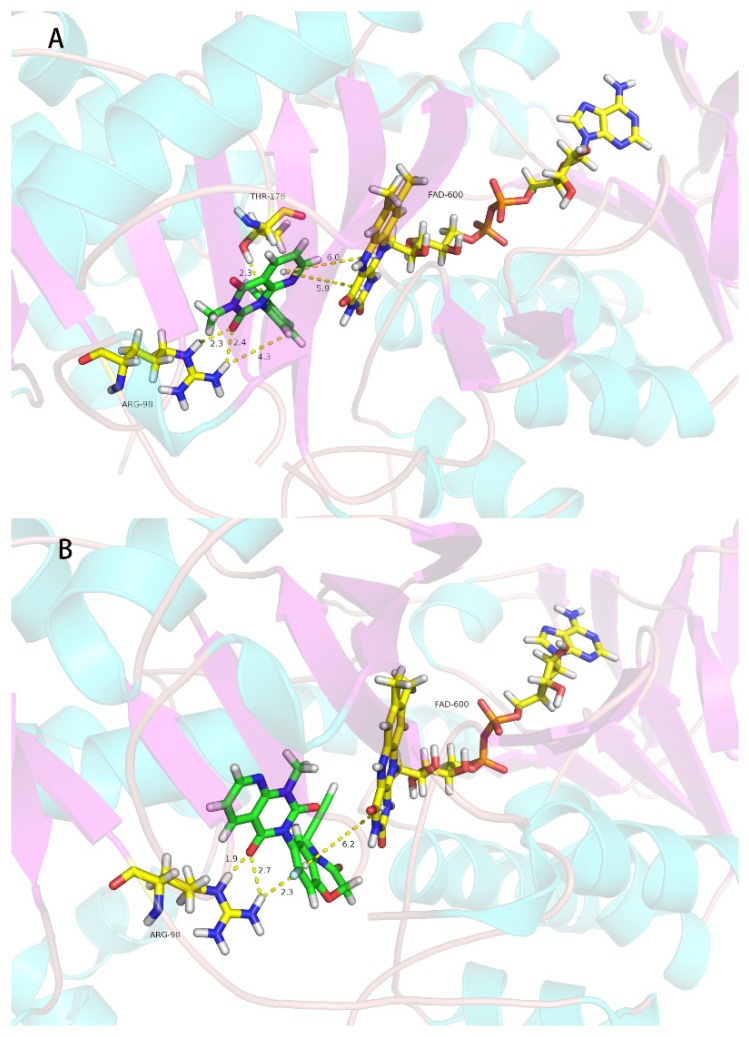

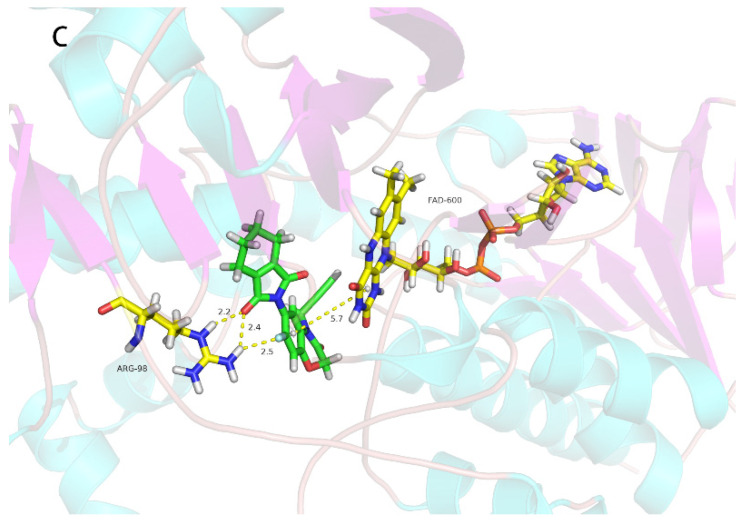
Simulated binding modes of compound **2o** (**A**), compound B (**B**) and flumioxazin (**C**) with NtPPO.

The pyrido[2,3-*d*]pyrimidine compounds in this study were designed from the reported PPO inhibitors [19]; the herbicidal activity of **2o** is good, so we conducted molecular modeling analysis of binding of this compound with *Nicotiana tabacum* PPO. As shown in Figure 4a, there were two π–π interactions between the pyrido[2,3-*d*]pyrimidine ring and FAD600 with the distances of 5.9 Å and 6.0 Å, respectively; second, there were three strong hydrogen bonding interactions (2.3 Å, 2.4 Å and 4.3 Å) between Agr98 of PPO and the F atom of 3-methyl-1-(2,3,4-trifluorophenyl)pyrido[2,3-*d*]pyrimidine-2,4(1*H*,3*H*)-dione (**2o**), and one hydrogen bonding interaction (2.3 Å) between Thr176 of PPO and the F atom of 3-methyl-1-(2,3,4-trifluorophenyl)pyrido[2,3-*d*]pyrimidine-2,4(1*H*,3*H*)-dione (**2o**), respectively. There were two π–π interactions (6.0 Å and 5.9 Å) between FAD and 3-methyl-1-(2,3,4-trifluorophenyl)pyrido[2,3-*d*]pyrimidine-2,4(1*H*,3*H*)-dione (**2o**). For the reported PPO inhibitor **B** [19], compound **B** also had three strong hydrogen bonds (1.9 Å, 2.7 Å and 2.3 Å) between the oxygen atom and fluorine atom of compound **B** and Arg98 of PPO. There was only one π–π interaction (6.2 Å) between FAD and compound **B**. Compared with the commercial PPO inhibitor flumioxazin, compound **B** had the same molecular interaction mode, but the commercialized PPO inhibitor flumioxazin has stronger effects than compound **B** in terms of hydrogen bonding or π–π stacking (short distance). Their different biological activity may be attributable to the different hydrogen bonds and π–π interactions. PPO is the molecular target of 22 commercial herbicides (Herbicide Resistance Action Committee, 2022) [33] with different weed and crop selectivities and different mechanisms of evolved weed resistances. In conclusion, the molecular docking study indicated that **2o** is a PPO inhibitor.

### 2.5. Electrolyte-Leakage Assay

**Figure 5:**
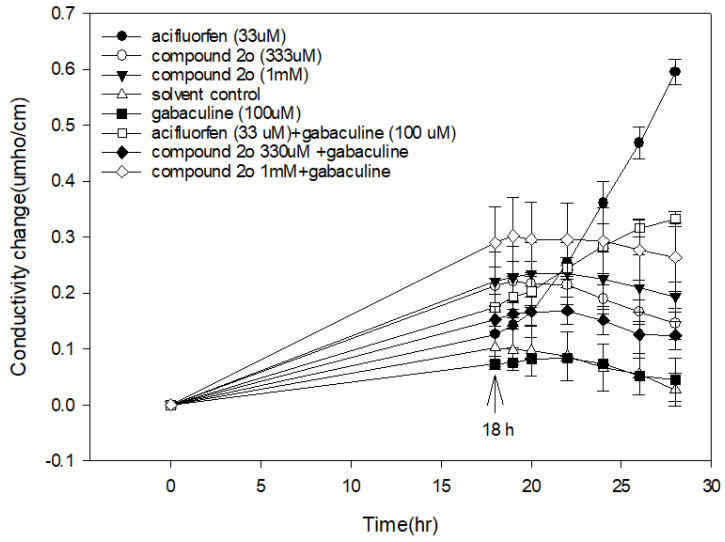
The electrolyte leakage of cucumber cotyledon discs caused by compound **2o** at different concentrations. The discs were in complete darkness for 18 h after which they were exposed to continuous light. Error bars are ±1 SE.

PPO inhibitors cause rapid loss of cellular electrolytes and bleaching of chloroplast pigments by the photodynamic action of protoporphyrin IX that accumulates when PPO is inhibited. This effect is easily shown in the light-dependent electrolyte-leakage method using the porphyrin pathway inhibitor gabaculine to reverse the effect [34]. Because the docking study indicated that **2o** is a PPO inhibitor, the same electrolyte-leakage assay was performed with it and the PPO inhibitor acifluorfen as a positive control. Acifluorfen caused light-dependent electrolyte leakage that was partially reversed by gabaculine (Figure 5).

### 2.6. DFT Calculation

**Figure 6:**
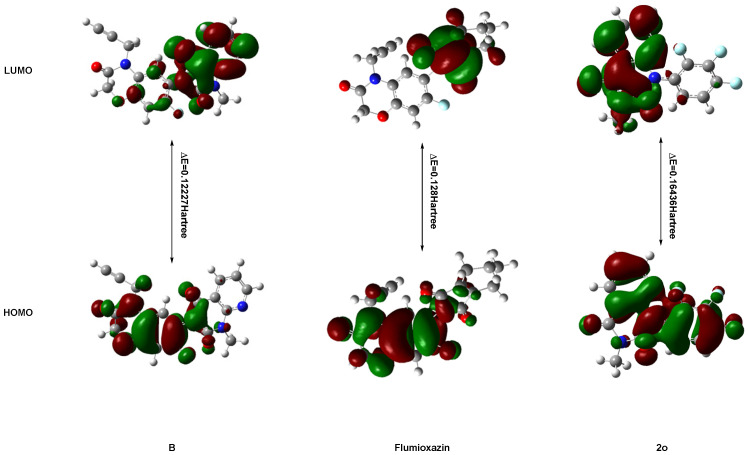
The HOMO and LUMO of compounds **2o**, **B** and flumioxazin.

Frontier molecular orbital (FMO) energy and molecular total energy of the highest active compound 3-methyl-1-(2,3,4-trifluorophenyl)pyrido[2,3-*d*]pyrimidine-2,4(1*H*,3*H*)-dione (**2o**) and lead compound **B** (Figure 1) are listed in Table 4 and shown in Figure 6. The energy gaps between HOMO and LUMO of the two compounds were also calculated with B3LYP.

According to the FMO theory, HOMO and LUMO are the most important physicochemical parameters that affect the bioactivity. The highest occupied molecular orbital (HOMO) provides electrons, and the lowest unoccupied molecular orbital (LUMO) accepts electrons. Thus, the study of the frontier orbital energy can provide useful information about the binding mechanism to a molecular target. From Figure 3, the geometry of the two compounds is divided into two parts: pyrido[2,3-*d*]pyrimidine-2,4(1*H*,3*H*)-dione ring and benzene ring. The HOMO of compound B is mainly located on the benzene ring and a little uracil ring, while the HOMO of compound **2o** is mainly located on the benzene ring and pyrido[2,3-*d*]pyrimidine-2,4(1*H*,3*H*)-dione ring. The LUMO of compound **B** is mainly located on the pyrido[2,3-*d*]pyrimidine-2,4(1*H*,3*H*)-dione ring, while the LUMO of compound **2o** is mainly located on the pyrido[2,3-*d*]pyrimidine-2,4(1*H*,3*H*)-dione ring. For the lead compound **B**, the electron transition from the benzene ring to the pyrido[2,3-*d*]pyrimidine-2,4(1*H*,3*H*)-dione ring and the energy gap between the HOMO and LUMO are 0.12227 Hartree. For the highest active compound, 3-methyl-1-(2,3,4-trifluorophenyl)pyrido[2,3-*d*]pyrimidine-2,4(1*H*,3*H*)-dione (**2o**), the electron transition from the pyrido[2,3-*d*]pyrimidine-2,4(1*H*,3*H*)-dione ring to the benzene ring and the energy gap between the HOMO and LUMO are 0.16463 Hartree. The total energies of the two compounds are different: lead compound **B** (−1351.05151151 Hartree) is lower than compound **2o** (−1152.14132348 Hartree). Also, the ClogP values of the two compounds differ with the ClogP values of **B** and **2o** at 2.69413 and 3.07961, respectively. This also implies that the benzene ring and methyl group were exchanged, which had an important impact on the activity by which the compound with lower total energy, lower energy gap and ClogP will exhibit good activity. The combination of MO results provided meaningful clues as to the structural features of this new family of herbicides that will be helpful in the future design of more potent compounds.

### 2.7. Molecular Structure Comparisons

The herbicidal activity and its interaction with the target enzyme PPO are greatly affected by their properties. The similar property principle (SPP) is that, when molecules interact with a target protein [35,36], small molecules have similar structures, properties and binding modes. In this paper, the most active compound **2o** and the reported herbicidal active compound **B** were selected as the structures for comparison. By comparing the Lipinski properties of compound **2o**, compound **B** and the PPO-inhibiting herbicide flumioxazin (Table 5), it is obvious that it is similar to the aromatic rings (ARs) of compound **2o** and compound **B**, which is more than that of the positive control flumioxazin. While for the rotatable bonds (RBs), it is similar to compound **B** and flumioxazin, which is more than that of compound **2o**. Furthermore, the molecular weight (MW) and hydrogen-bond acceptors (HBAs) of compound **2o** were lower than those of compound **B** and flumioxazin, which may not be conducive to the metabolism of exogenous compounds for the plant. Only hydrogen-bond donors (HBDs) were found in the three compounds, which is the same (no HBDs).

### 2.8. Absorption, Distribution, Metabolism, Excretion and Toxicity (ADMET) Prediction

Many pesticides have failed to enter the market due to their toxicity. The pharmacokinetic properties and potential Ames test results for mutagenicity of the active compound **2o**, compound **B** and flumioxazin are predicted in Table 6. The solubility level, absorption level, CYP2D6 prediction, plasma protein binding and Ames test results of the three compounds were very similar. The Ames test prediction indicated that the three compounds are non-mutagenic.

## 3. Materials and Methods

### 3.1. Instruments

Melting points were determined using an X-4 apparatus and were uncorrected. ^1^H NMR and ^13^C NMR spectra were measured on a Bruker AC-P500 or AV-400 instrument using TMS as an internal standard and CDCl_3_ as the solvent. HRMS was determined on a JEOL AccuTOF (JMS-T 100LC) instrument equipped with both DART and electrospray ionization (ESI) ion sources. All the reagents were of analytical grade or freshly prepared before use.

### 3.2. Synthesis

The novel pyrido[2,3-*d*]pyrimidine compounds were synthesized according to the route shown in Scheme 1, and the yields were not optimized.

#### General Synthesis of Target Compound **2**

The starting materials 1 were synthesized in our lab [32].

To a solution of starting materials 1 (1 mmol) in DMF (30 mL), NaH (0.053 g, 2.2 mmol) was added in batches at low temperature. Then, the solution was refluxed. After that, methyl iodide (0.14 g, 1.1 mmol) was added dropwise to the mixture, TLC monitor. Then, the mixture was cooled and H_2_O was added; the mixture was extracted with EA, washed with a saturated NaCl solution and dried with Na_2_SO_4_, and the solvent was removed to obtain a crude product. The crude product was separated and purified with silica gel column chromatography to obtain the target product **2a**~**2o**.

*1-(3,5-dimethylphenyl)-3-methylpyrido[2,3-d]pyrimidine-2,4(1H,3H)-dione* **2a**

Light yellow solid, yield 84%, m.p. 246–248 °C, ^1^H NMR (CDCl_3_, 500 MHz), δ: 2.40 (s, 6H, CH_3_), 3.54 (s, 3H, CH_3_), 6.94 (s, 2H, Ph), 7.15 (s,1H, Ph), 7.21–7.23 (m, 1H, Py), 8.52–8.54 (m, 1H, Py), 8.56–8.58 (m, 1H, Py); ^13^C NMR (101 MHz, CDCl_3_) δ 161.52, 154.40, 151.90, 151.28, 139.42, 137.61, 135.64, 131.04, 126.46, 119.20, 110.48, 28.55, 21.37; HRMS (ESI) *m*/*z*: Calculated, 282.1237, Found, 282.1242 [M + H]^+^.

*1-(2,6-dichlorophenyl)-3-methylpyrido[2,3-d]pyrimidine-2,4(1H,3H)-dione* **2b**

Light yellow solid, yield 82%, m.p. 233–234 °C, ^1^H NMR (CDCl_3_, 500 MHz), δ: 3.57 (s, 3H, CH_3_), 7.27–7.29 (m, 1H, Py), 7.43 (t, *J* = 6.4 Hz, 1H, Ph), 7.54 (d, *J* = 6.8 Hz, 2H, Ph), 8.52–8.53 (m, 1H, Py), 8.55–8.57 (m, 1H, Py); ^13^C NMR (101 MHz, CDCl_3_) δ 161.39, 154.49, 150.15, 149.44, 137.87 (C2), 135.25, 131.87 (C2), 130.78, 128.72, 119.90, 110.39, 28.62; HRMS (ESI) *m*/*z*: Calculated, 322.0145, Found, 322.0145 [M + H]^+^.

*1-(3,4-difluorophenyl)-3-methylpyrido[2,3-d]pyrimidine-2,4(1H,3H)-dione* **2c**

Light yellow solid, yield 79%, m.p. 191–193 °C, ^1^H NMR (CDCl_3_, 500 MHz), δ: 3.54 (s, 3H, CH_3_), 7.08–7.11 (m, 1H, Ph), 7.17–7.21 (m, 1H, Ph), 7.25–7.28 (m, 1H, Py), 7.34–7.37 (m, 1H, Py), 8.52–8.54 (m, 2H, Py-Ph); ^13^C NMR (101 MHz, CDCl_3_) δ 161.16, 154.16, 151.32, 150.92, 150.60 (dd, *J* = 256.0, 1.7 Hz), 150.47 (dd, *J* = 251.9, 2.9 Hz), 137.85, 131.69 (dd, *J* = 8.1, 3.9 Hz), 125.68 (dd, *J* = 6.7, 3.8 Hz), 119.71, 118.95 (dd, *J* = 18.7, 1.0 Hz), 117.95 (dd, *J* = 18.5, 0.9 Hz), 110.56, 28.58; HRMS (ESI) *m*/*z*: Calculated, 290.0736, Found, 290.0736 [M + H]^+^.

*1-(4-chlorophenyl)-3-methylpyrido[2,3-d]pyrimidine-2,4(1H,3H)-dione* **2d**

Light yellow solid, yield 82%, m.p. 240–242 °C, ^1^H NMR (CDCl_3_, 500 MHz), δ: 3.54 (s, 3H, CH_3_), 7.24–7.26 (m, 1H, Py), 7.27 (d, *J* = 6.8 Hz, 2H, Ph), 7.55 (d, *J* = 6.8 Hz, 2H, Ph), 8.54–8.56 (m, 2H, Py); ^13^C NMR (101 MHz, CDCl_3_) δ 161.28, 154.18, 151.50, 150.98, 137.78, 134.92, 134.24, 130.43 (C2), 129.89 (C2), 119.53, 110.58, 28.58; HRMS (ESI) *m*/*z*: Calculated, 288.0534, Found, 288.0534 [M + H]^+^.

*3-methyl-1-phenylpyrido[2,3-d]pyrimidine-2,4(1H,3H)-dione* **2e**

Light yellow solid, yield 83%, m.p. 247–248 °C, ^1^H NMR (CDCl_3_, 500 MHz), δ: 3.55 (s, 3H, CH_3_), 7.22–7.25 (m, 1H, Py), 7.33–7.34 (m, 2H, Ph), 7.52–7.55 (m, 1H, Py), 7.58–7.61 (m, 2H, Ph), 8.54–8.56 (m, 2H, Py-Ph); ^13^C NMR (101 MHz, CDCl_3_) δ 161.46, 154.26, 151.76, 151.16, 137.64, 135.84, 129.63, 129.01, 128.98, 119.30, 110.52, 28.55; HRMS (ESI) *m*/*z*: Calculated, 254.0924, Found, 254.0942 [M + H]^+^.

*3-methyl-1-(2-methyl-3-nitrophenyl)pyrido[2,3-d]pyrimidine-2,4(1H,3H)-dione* **2f**

Light yellow solid, yield 68%, m.p. 250–251 °C, ^1^H NMR (CDCl_3_, 500 MHz), δ: 2.21 (s, 3H, CH_3_), 3.54 (s, 3H, CH_3_), 7.28 (t, *J* = 6.2 Hz,1H, Ph), 7.40 (d, *J* = 6.8 Hz, 1H, Ph), 8.23–8.25 (m, 1H, Py), 8.30 (d, *J* = 5.0 Hz,1H, Ph), 8.49–8.50 (m, 1H, Py), 8.56–8.58 (m, 1H, Py); ^13^C NMR (101 MHz, CDCl_3_) δ 161.22, 154.46, 151.04, 150.89, 150.45, 138.03, 137.10, 134.10, 132.77, 127.36, 125.41, 119.91, 110.54, 28.66, 14.30; HRMS (ESI) *m*/*z*: Calculated, 313.0931, Found, 313.0944 [M + H]^+^.

*3-methyl-1-(o-tolyl)pyrido[2,3-d]pyrimidine-2,4(1H,3H)-dione* **2g**

Light yellow solid, yield 65%, ^1^H NMR (CDCl_3_, 500 MHz), δ: 2.11 (s, 3H, CH_3_), 3.56 (s, 3H, CH_3_), 7.21–7.25 (m, 2H, Ph), 7.39–7.44 (m, 3H, Py-Ph), 8.54–8.56 (m, 2H, Py); ^13^C NMR (101 MHz, CDCl_3_) δ 161.59, 154.58, 151.37, 150.64, 137.71, 136.42, 134.98, 131.24, 129.42, 128.88, 127.30, 119.30, 110.42, 28.57, 17.65; HRMS (ESI) *m*/*z*: Calculated, 268.1081, Found, 268.1083 [M + H]^+^.

*1-(2,6-difluorophenyl)-3-methylpyrido[2,3-d]pyrimidine-2,4(1H,3H)-dione* **2h**

Light yellow solid, yield 76%, m.p. 200–203 °C, ^1^H NMR (CDCl_3_, 500MHz), δ: 3.56 (s, 3H, CH_3_), 7.12–7.15 (m, 2H, Ph), 7.28–7.30 (m, 1H, Py), 7.48–7.52 (m, 1H, Ph), 8.54–8.56 (m, 2H, Py); ^13^C NMR (101 MHz, CDCl_3_) δ 161.23, 159.25 (dd, *J* = 253.0, 4.2 Hz) (C2), 154.34, 150.34, 149.77, 137.79, 130.86 (t, *J* = 9.9 Hz), 119.94, 112.08 (dd, *J* = 16.4, 2.0 Hz), (d, *J* = 23.5 Hz) (C2), 110.58, 38.65; HRMS (ESI) *m*/*z*: Calculated, 290.0736, Found, 290.0744 [M + H]^+^.

*1-(2-chlorophenyl)-3-methylpyrido[2,3-d]pyrimidine-2,4(1H,3H)-dione* **2i**

Light yellow solid, yield 82%, m.p. 243–245 °C, ^1^H NMR (CDCl_3_, 500 MHz), δ: 3.56 (s, 3H, CH_3_), 7.24–7.27 (m,1H, Py), 7.40–7.42 (m, 1H, Ph), 7.49–7.51 (m, 2H, Ph), 7.62–7.64 (m, 1H, Ph), 8.53–8.57 (m, 2H, Py); ^13^C NMR (101 MHz, CDCl_3_) δ 161.43, 154.36, 151.06, 150.37, 137.73, 133.67, 133.36, 130.95, 130.52, 130.45, 128.03, 119.57, 110.43, 28.58; HRMS (ESI) *m*/*z*: Calculated, 288.0534, Found, 288.0545 [M + H]^+^.

*1-(2,5-dimethoxyphenyl)-3-methylpyrido[2,3-d]pyrimidine-2,4(1H,3H)-dione* **2j**

Light yellow solid, yield 81%, m.p. 158–160 °C, ^1^H NMR (CDCl_3_, 500 MHz), δ: 3.23 (s, 3H, CH_3_), 3.81 (s, 3H, CH_3_), 3.90(s, 3H, CH_3_), 6.61–6.63 (m,1H, Ph), 6.84 (d, *J* = 6.8 Hz, 1H, Ph), 7.39–7.42 (m, 1H, Py), 7.74–7.75 (m, 1H, Py), 8.05 (s, 1H, Ph), 8.52–8.54 (m, 1H, Py); HRMS (ESI) *m*/*z*: Calculated, 314.1135, Found, 314.1153 [M + H]^+^.

*3-methyl-1-(4-(trifluoromethyl)phenyl)pyrido[2,3-d]pyrimidine-2,4(1H,3H)-dione* **2k**

Light yellow solid, yield 81%, m.p. 225–227 °C, ^1^H NMR (CDCl_3_, 500 MHz), δ: 3.55 (s, 3H, CH_3_), 6.61–6.63 (m,1H, Ph), 7.26–7.28(m, 1H, Py), 7.47(d, *J* = 6.6 Hz, 2H, Ph), 7.84(d, J = 6.6 Hz, 2H, Ph), 8.52–8.53 (m, 1H, Py), 8.56–8.57(m, 1H, Py); ^13^C NMR (101 MHz, CDCl_3_) δ 161.22, 154.13, 151.28, 150.84, 138.94, 137.87 (C2), 131.01 (d, *J* = 32.8 Hz), 129.78, 126.73 (q, *J* = 3.8 Hz), 123.73 (q, *J* = 272.5 Hz), 119.71 (C2), 110.60, 28.59; HRMS (ESI) *m*/*z*: Calculated, 322.0798, Found,322.0820[M + H]^+^.

*1-(3-chlorophenyl)-3-methylpyrido[2,3-d]pyrimidine-2,4(1H,3H)-dione* **2l**

Light yellow solid, yield 78%, m.p. 232–234 °C, ^1^H NMR (CDCl_3_, 500 MHz), δ: 3.55 (s, 3H, CH_3_), 7.26–7.28 (m,2H, Ph), 7.33–7.35 (m,1H, Ph), 7.45–7.47 (m, 1H, Py), 7.63 (s, 1H, Ph), 8.52–8.53 (m, 1H, Py), 8.54–8.56 (m, 1H, Py); ^13^C NMR (101 MHz, CDCl_3_) δ 161.27, 154.22, 151.38, 150.90, 137.77, 136.78, 135.04, 130.46, 129.60, 129.33, 127.44, 119.59, 110.51, 28.57; HRMS (ESI) *m*/*z*: Calculated, 288.0534, Found, 288.0541 [M + H]^+^.

*1-(2,4-dichlorophenyl)-3-methylpyrido[2,3-d]pyrimidine-2,4(1H,3H)-dione* **2m**

Light yellow solid, yield 85%, m.p. 231–232 °C, ^1^H NMR (CDCl_3_, 500 MHz), δ: 3.55 (s, 3H, CH_3_), 7.26–7.28 (m,1H, Ph), 7.34 (d, *J* = 6.4 Hz,1H, Ph), 7.45–7.47 (m, 1H, Py), 7.63 (s, 1H, Ph), 8.52–8.53 (m, 1H, Py), 8.54–8.56 (m, 1H, Py); ^13^C NMR (101 MHz, CDCl_3_) δ 161.31, 154.35, 150.87, 150.25, 137.89, 135.86, 134.35, 132.37, 131.82, 130.48, 128.48, 119.81, 110.51, 28.65; HRMS (ESI) *m*/*z*: Calculated, 322.0145, Found, 322.0154 [M + H]^+^.

*1-(2,6-diethylphenyl)-3-methylpyrido[2,3-d]pyrimidine-2,4(1H,3H)-dione* **2n**

Light yellow solid, yield 83%, m.p. 184–186 °C, ^1^H NMR (CDCl_3_, 500 MHz), δ: 1.10 (t, *J* = 6.0 Hz, 6H, CH_3_), 2.27–2.43 (m,4H,CH_2_), 3.57 (s, 3H, CH_3_), 7.22–7.24 (m, 1H, Py), 7.30 (d, *J* = 6.1 Hz, 2H, Ph), 7.46 (t, *J* = 6.1 Hz, 1H, Ph), 8.54–8.57 (m, 2H, Py); ^13^C NMR (101 MHz, CDCl_3_) δ 161.74, 154.79, 151.50, 150.54, 141.41, 137.73 (C2), 132.97, 129.64 (C2), 126.58, 119.38, 110.33, 28.67, 24.14 (C2), 13.67 (C2); HRMS (ESI) *m*/*z*: Calculated, 310.1550, Found, 310.1532 [M + H]^+^.

*3-methyl-1-(2,3,4-trifluorophenyl)pyrido[2,3-d]pyrimidine-2,4(1H,3H)-dione* **2o**

Light yellow solid, yield 85%, m.p. 180–182 °C, ^1^H NMR (CDCl_3_, 500 MHz), δ: 3.55 (s, 3H, CH_3_), 7.14–7.17 (m, 2H, Ph), 7.28–7.31 (m, 1H, Py), 8.53–8.56 (m, 2H, Py); ^13^C NMR (101 MHz, CDCl_3_) δ 161.07, 154.24, 151.56 (ddd, *J* = 252.5, 10.0, 3.0 Hz), 150.60, 150.31, 148.29 (ddd, *J* = 254.7, 10.7, 3.8 Hz), 140.69 (ddd, *J* = 253.4, 15.9, 14.0 Hz), 137.90, 124.89 (dd, *J* = 8.1, 3.9 Hz), 120.71 (dd, *J* = 11.0, 4.0 Hz), 120.02, 112.15 (dd, *J* = 18.5, 3.9 Hz), 110.55, 28.62; HRMS (ESI) *m*/*z*: Calculated, 308.0641, Found, 308.0618 [M + H]^+^.

### 3.3. Crystal Determination

The crystal of compound **2n** (0.52 mm × 0.46 mm × 0.42 mm) was mounted on a Bruker ‘CCD area detector’ diffractometer with MoKα radiation (λ = 0.71073Å) by using a phi and scan modes at 296(2) K in the range of 5.382° ≤ θ ≤ 60.004°. The compound **2n** belongs to a monoclinic system with space group P2_1_/c and parameters of a = 8.821(5)Å, b = 12.379(7)Å, c = 15.399(8)Å, α = 90°, β = 100.522(18)°, γ = 90°, V = 1653.2(16) Å^3^, Z = 4, μ(MoKα) = 0.083 mm^−1^ and Dcalc = 1.243 g/cm^3^. Using Olex2 [37], the structure was solved with the ShelXS [38] structure solution program using Direct Methods and refined with the ShelXL [39] refinement package using Least Squares minimization. The detailed information is in the Supplementary Materials.

### 3.4. Herbicide Bioassays

#### 3.4.1. Bioassays with Lettuce and Bentgrass

The phytotoxicity of the pyrido[2,3-*d*]pyrimidine compounds **2a**~**2o** was tested on bentgrass (*Agrostis stolonifera*) and lettuce (*Lactuca sativa*) in a bioassay used previously [30]. Briefly, seeds of bentgrass and lettuce were surface-sterilized for 10 min with a 0.5−1% (*v*/*v*) solution of NaOCl, rinsed with deionized H_2_O and then dried in a semi-sterile environment. A 1.5 cm Whatman Grade 1 filter paper disc was kept on a 24-well plate. Every well contained 200 μL of the control solution (deionized H_2_O, 200 μL), control solvent (deionized H_2_O, 180 μL + test compound solvent, 20 μL) or samples (deionized H_2_O, 180 μL + sample, 20 μL). Deionized H_2_O was added into the wells before the solvent or samples. Technical-grade commercial herbicides (clomazone and flumioxazin) were used as the positive controls. The samples were dissolved in CH_3_COCH_3_. Ten milligrams of bentgrass seeds or five lettuce seeds were added into each 24-well plate for the bioassays, and then, the plate was sealed using Parafilm. These 24-well plates were incubated ((bentgrass, 12 days) and (lettuce, 5 days)) in a CU-36L5 Percival Scientific (Perry, IA, USA) incubator. The temperature and light conditions were continuous light (120 μmol·s^−1^·m^−2^) at 26 °C. The phytotoxicity of the control solvent or samples was quantitated as 0−5 grade: 5 is no germination of the seeds, 4 is more than 50% germination of the seeds, 3 is approximately 50% germination of the seeds, 2 is less than 50% germination of the seeds, 1 is some stunting to the seedlings but no inhibition of germination, and 0 is no effect. Each experiment was repeated twice.

#### 3.4.2. Bioassays with Field Mustard and Wheat

##### Inhibition of the Root Growth of Field Mustard (*Brassica campestris*)

The evaluated compounds were dissolved in water and emulsified if necessary. Field mustard seeds were soaked in distilled water for 4 h before being placed on a filter paper in a 6 cm Petri plate to which 2 mL of inhibitor solution had been added in advance. Usually, 15 seeds were used on each plate. The plate was placed in a dark room and allowed to germinate for 65 h at 28 ± 1 °C. The lengths of 10 field mustard roots selected from each plate were measured, and the means were calculated. The control was carried out in distilled water only. The inhibition rates were calculated from the root length.

##### Inhibition of the Seedling Growth of Wheat (*Triticum aestivum*)

The evaluated compounds were dissolved in water and emulsified if necessary. Ten wheat seeds were placed into a 50 mL cup covered with a layer of glass beads and a piece of filter paper at the bottom to which 5 mL of inhibitor solution had been added in advance. The cup was placed in a brightly lighted room and allowed to germinate for 65 h at 28 ± 1 °C. The heights of the seedlings of the above-ground plant parts from each cup were measured, and the means were calculated. The control was carried out in distilled water only. The inhibition rates were calculated from the plant heights.

### 3.5. Electrolyte Leakage

The effect of pure compound **2o** on plasma membrane stability was determined on cucumber cotyledon discs using a previously published method [34]. Under dim green light, the cucumber cotyledon discs (4 mm) were cut, and 50 discs were floated in Petri plates (sterile, 6 × 1.5 cm, polystyrene) containing 4850 μL of 1 μM MES buffer (pH = 6.5) with 2% sucrose plus CH_3_COCH_3_ (150 μL) or test sample solution (150 μL). Compound **2o** (333 and 1 mM), the positive control acifluorfen and a solvent control (CH_3_COCH_3_) were tested three times. Acifluorfen causes rapid plasma-membrane leakage in the light due to its activity as a protoporphyrinogen oxidase (PPO) inhibitor. At the beginning of the experiment, 55 discs were placed in test tubes with buffer (5 mL). These tubes were placed in boiling H_2_O for 15 min and then cooled to 20 °C, after which electrical conductivity was determined at 0 h. Then, the dishes were placed under 18 h dark incubation. After that, the electrical conductivity was determined at 19, 20, 22 and 24 h under a high light intensity condition. Graphs of electrical conductivity vs. time were drawn from these data.

### 3.6. Porphyrin-Dependent Activity

To determine if the leakage caused by **2o** is due to production of a porphyrin, the experiment [34] was conducted in which the porphyrin synthesis inhibitor gabaculine was used with the herbicide. The PPO inhibitor acifluorfen was used as a positive control. Gabaculine itself has no significant effect in this bioassay. A reduction in the herbicide-caused electrolyte leakage by gabaculine occurs with PPO inhibitors such as acifluorfen.

### 3.7. Molecular Docking

The molecular docking simulations were carried out to analyze the interaction mode using Discovery Studio 2.5 software [40] according to the reported method [41]. The structure of *Nicotiana tabacum* PPO [42] was downloaded from the protein data bank (PDB, https://www.rcsb.org/, ID: 1SEZ). The protein crystal structure of *Nt*PPO and the small molecule 3-methyl-1-(2,3,4-trifluorophenyl)pyrido[2,3-*d*]pyrimidine-2,4(1*H*,3*H*)-dione (**2o**) were prepared with standard methods using Discovery Studio 2.5 software. After molecular docking, the best binding mode was selected according to the results of docking energy compared with the ligand in the *Nt*PPO.

### 3.8. Density Functional Theory Analysis (DFT Analysis)

The most active compound, 3-methyl-1-(2,3,4-trifluorophenyl)pyrido[2,3-*d*]pyrimidine-2,4(1*H*,3*H*)-dione (**2o**) (Table 2), and compound B (Figure 1) were selected and drawn in Gaussview 5.0. The two structures were optimized using DFT-B3LYP/6-31G methods in the Gaussian 03 package [43]. Vibration analysis indicated that the two optimized structures were in accordance with the minimum points on the potential energy surfaces, which means no virtual frequencies, proving that the obtained optimized structures were stable. All the convergent precisions were the system default values.

## 4. Conclusions

In conclusion, a series of pyrido[2,3-*d*]pyrimidine compounds were synthesized using a one-pot method with NaH as the base. This method avoids using expensive catalysts, and the reaction works well and efficiently. The herbicidal activity of the synthesized compounds against lettuce and bentgrass was tested at 1 mM, and some of the compounds showed good herbicidal activity against the monocot bentgrass but not against the dicot lettuce. The most active compound against lettuce and bentgrass was the most active experimental compound in inhibition of wheat shoot growth with activity almost the same as flumioxazin. Molecular binding studies indicate that the most active compound is a PPO inhibitor. However, this compound does not cause light-dependent cellular leakage of cucumber cotyledon discs that can be reversed by an inhibitor of porphyrin synthesis. The likely explanation for this is that the compound is either not taken up and/or metabolically altered to a compound with no effect on PPO by cucumber cotyledon discs.

## Figures and Tables

**Table 1:** The effect of temperature on the formation of compound **2d**.

Entry	Solvent	Time/h	Temperature/°C	Yield
**1**	THF	4	0	0%
**2**	THF	4	r.t.	15%
**3**	THF	24	r.t.	21%
**4**	THF	4	50	70%
**5**	DMF	8	40	56%
**6**	DMF	8	50	82%
**7**	DMF	8	55	75%

**Table 2:** The herbicidal activities of compounds **2a**–**2o** (1 mM) against two plant species. No effect = 0, and complete inhibition of germination = 5.

Compound	R	Lettuce	Bentgrass
**2a**	3,5-di-CH_3_	0	3
**2b**	2,6-di-Cl	0	3
**2c**	3,4-di-F	1	4
**2d**	4-Cl	1	4
**2e**	H	1	4
**2f**	2-CH_3_-3-NO_2_	1	4
**2g**	2-CH_3_	1	4
**2h**	2,6-di-F	0	4
**2i**	2-Cl	0	4
**2j**	2,5-di-OCH_3_	0	2
**2k**	4-CF_3_	0	3
**2l**	3-Cl	0	4
**2m**	2,4-di-Cl	2	4
**2n**	2,6-di-CH_2_CH_3_	1	4
**2o**	2,3,4-tri-F	1	5
Clomazone		5	5
Flumioxazin		5	5

**Table 3:** Herbicidal activities (inhibitory, %) of target compounds **2a**~**2o**.

No.	Root Length of Field Mustard Seedlings		Shoot Length of Wheat Seedlings	
	100 ppm	10 ppm	100 ppm	10 ppm
**2a**	53.9	43.5	28.6	3.4
**2b**	12.8	0	51.4	21.7
**2c**	45.3	17.6	72.6	55.9
**2d**	47.3	21.4	67.6	42.4
**2e**	68.5	34.1	32.1	4.1
**2f**	33.9	26.1	37.9	−2.1
**2g**	39.9	14.5	64.6	32.1
**2h**	12.2	0	77.3	52.0
**2i**	14.8	0	62.3	45.7
**2j**	15.6	0	63.5	27.8
**2k**	73.5	38.3	31.4	3.2
**2l**	14.1	0	59.8	38.5
**2m**	18.5	0	49.8	21.3
**2n**	32.7	12.3	73.2	51.7
**2o**	46.6	25.7	89.7	78.3
Water	0	0	0	0
Flumioxazin	75.0	59.8	100	94.3

**Table 4:** Total energy and frontier orbital energy of two related compounds that inhibit PPO.

Energy	2o	B (Figure 1)	Flumioxazin
*E*_total_/Hartree ^b^	−1152.14132348	−1351.05151151	−1242.88439254
*E*_HOMO_/Hartree	−0.23915	−0.20471	−0.22754
*E*_LUMO_/Hartree	−0.07452	−0.08244	−0.09954
Δ*E* ^a^/Hartree	0.16463	0.12227	0.128
CLogP	3.07961	2.69413	2.2541
tPSA	52.98	82.52	66.92

^a^: Δ*E = E*_LUMO_-*E*_HOMO_; ^b^: 1 hartree = 2625.5 kJ mol⁻¹.

**Table 5:** Lipinski property comparisons of compound **2o**, B and flumioxazin.

	2o	B	Flumioxazin
Structures	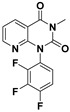	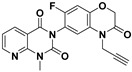	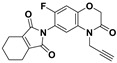
Interaction energy ^a^ (kcal/mol)	28.4152	43.7645	36.2907
MW ^a^	307.227	380.329	354.332
HBAs ^a^	3	5	4
HBDs ^a^	0	0	0
RBs ^a^	1	2	2
ARs ^a^	2	2	1

^a^ Discovery Studio 3.5 for interaction energy, MW, rotatable bonds (RBs) and aromatic rings (ARs).

**Table 6:** ADMET prediction of compound **2o**, B and flumioxazin.

	2o	B	Flumioxazin
solubility level ^a^	2	2	2
absorption level ^b^	0	0	0
CYP2D6 prediction ^c^	false	false	false
AlogP98 ^d^	3.082	2.955	3.835
PPB# prediction ^e^	true	true	true
Ames	Non-Mutagen	Non-Mutagen	Non-Mutagen

^a^ Solubility level: categorical solubility level. 2: Yes, low; 4: Yes, optimal ^b^ Absorption level: absorption level. 0: Good absorption. ^c^ CYP2D6: cytochrome P450 2D6. <0.161: False, noninhibitor; >0.161: true, inhibitor. ^d^ AlogP98: the logarithm of the partition coefficient between n-octanol and water. <4.0: Binding is <90%; >4.0: binding is >90% and binding is <95%. ^e^ PPB: plasma protein binding ability. <−2.209: ≥90%, False; >−2.209: ≤90%, True.

## Data Availability

Data is contained within the article or Supplementary Materials.

## Supplementary Materials

The following supporting information can be downloaded at: https://www.mdpi.com/article/10.3390/molecules28217363/s1.
