# Supplementary material for: Synthesis, Herbicidal Activity, Mode of Action, and In Silico Analysis of Novel Pyrido[2,3-d]pyrimidine Compounds
Source: Molecules. 2023 Oct 31;28(21):7363. doi: 10.3390/molecules28217363 (PMC10647610; doi:10.3390/molecules28217363)
Supplement: Supplementary file 1 [file molecules-28-07363-s001.zip › molecules-2690855-supplementary.pdf]

Supplemental Materials

Synthesis, Herbicidal Activity, Mode of Action and In Silico Analysis of Novel Pyrido [2,3-*d*]pyrimidine Compounds

Li-Jing Min[1†], Wei Liang[2†], Joanna Bajsa-Hirschel[3†], Peng Ye[4†], Qiao Wang[2], Xin-Peng Sun[2,5], Charles L. Cantrell[3], Liang Han[2], Na-Bo Sun[5*], Stephen O. Duke[6*], Xing-Hai Liu[2*]

1 College of Life Science, Key Laboratory of Vector Biology and Pathogen Control of Zhejiang Province, Huzhou University, Huzhou, 313000, Zhejiang, China, 178341894@qq.com (L.J.)

2 College of Chemical Engineering, Zhejiang University of Technology, Hangzhou, 310014, Zhejiang, China; 1907689855@qq.com (W.L.); 751716045@qq.com (Q.W.); xhliu@zjut.edu.cn (X.L.); han-liang@zjut.edu.cn(L.H.)

3 Natural Products Utilization Research Unit, Agricultural Research Service, U.S. Department of Agri-culture, P.O. Box 1848, University, Mississippi 38677, USA, charles.cantrell@usda.gov (C.C.); joan-na.bajsa-hirsche@usda.gov (J.B.)

4 Shanghai Souguo Science & Technology Co. Ltd., Shanghai, 201708, China, 52316676@qq.com(P.Y.)

5 College of Biology and Environmental Engineering, Zhejiang Shuren University, Hangzhou 310015, Zhejiang, China, nabosun@126.com (N.B.)

6 National Center for Natural Product Research, School of Pharmacy, University of Mississippi, P.O. Box 1848, University, Mississippi, 38677, USA, sduke@olemiss.edu (S.D.)

* Correspondence: nabosun@126.com; sduke@olemiss.edu; xhliu@zjut.edu.cn

Table of contents

1. The detailed bioassay method………..................... 2
2. The spectroscopy (^{1}H NMR, ^{13}C NMR and HRMS) of target compounds……….... 3-24
3. The crystal data of compound 2n......................... 25-30

1. herbicidal activity

Seeds of lettuce (*Lactuca sativa*—Iceberg A Crisphead cultivar from Burpee Seeds, Warminster, PA, USA) and bentgrass (*Agrostis stolonifera*—Penncross variety obtained from Turf-Seed, Inc. of Hubbard, OR, USA) were surface sterilized with a 0.5% to 1% (v/v) sodium hypochlorite solution for approximately 10 min, rinsed with deionized water and dried in a sterile environment. A filter paper disk (Whatman Grade 1, 1.5 cm) was placed in each well of a 24-well plate. The control wells contained 200 μL of deionized water. The control + solvent well contained 180 μL of water and 20 μL of the solvent. All sample wells contained 180 μL of water and 20 μL of the appropriate dilution of the sample. Water was pipetted into the well before the sample or solvent. Test samples were dissolved in acetone and the final concentration of acetone in the wells was 10%. For the bioassay five lettuce seeds or 10 mg of bentgrass seeds were placed in each well before sealing the plate with Parafilm. The plates were incubated either for five days (lettuce) or 12 days (bentgrass) in a Percival Scientific (Perry, IA, USA) CU-36L5 incubator under continuous light conditions at 26 °C and 120 $\mu mol \cdot s^{-1} \cdot m^{-2}$ average photosynthetically active radiation (PAR). A qualitative estimate of phytotoxicity was made by assigning a rating of 0 for no effect (sample well plants looked identical to the control + solvent well plants; seeds had germinated and resulting seedlings had grown normally), 2 for less than 50% germination inhibition, 3 for about 50% germination inhibition, 4 for more than 50% germination inhibition, and 5 for no germination of the seeds. Each experiment was repeated three times.

2. The spectroscopy of target compounds

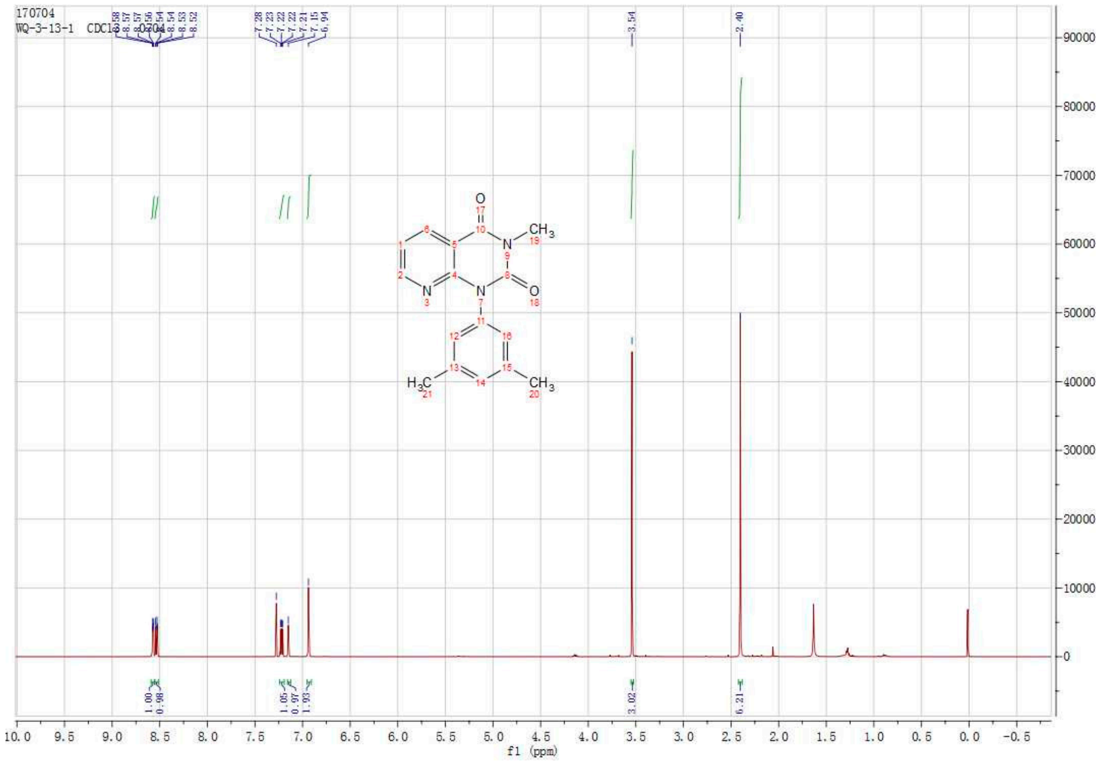

The ^{1}H NMR of compound 2a

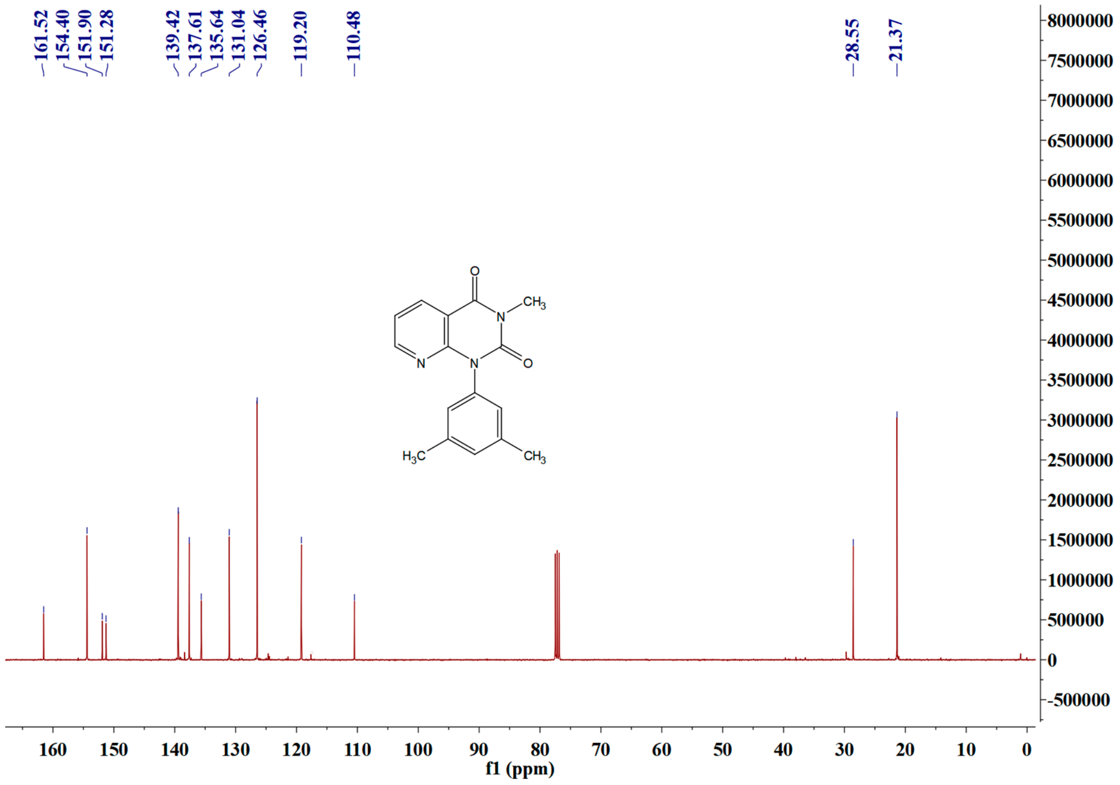

The ^{13}C NMR of compound 2a

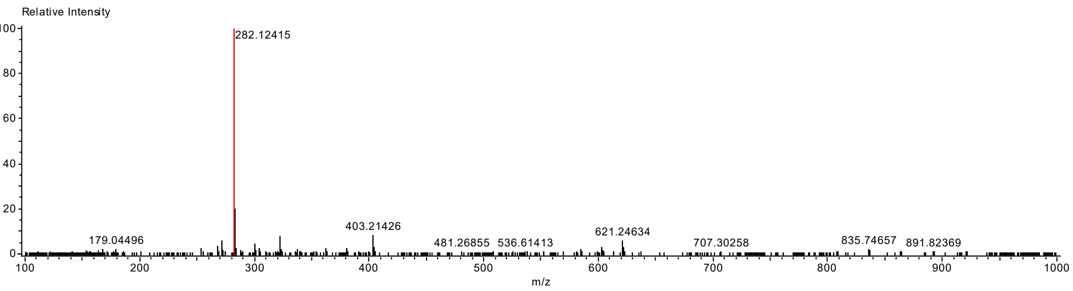

The HRMS of compound 2a

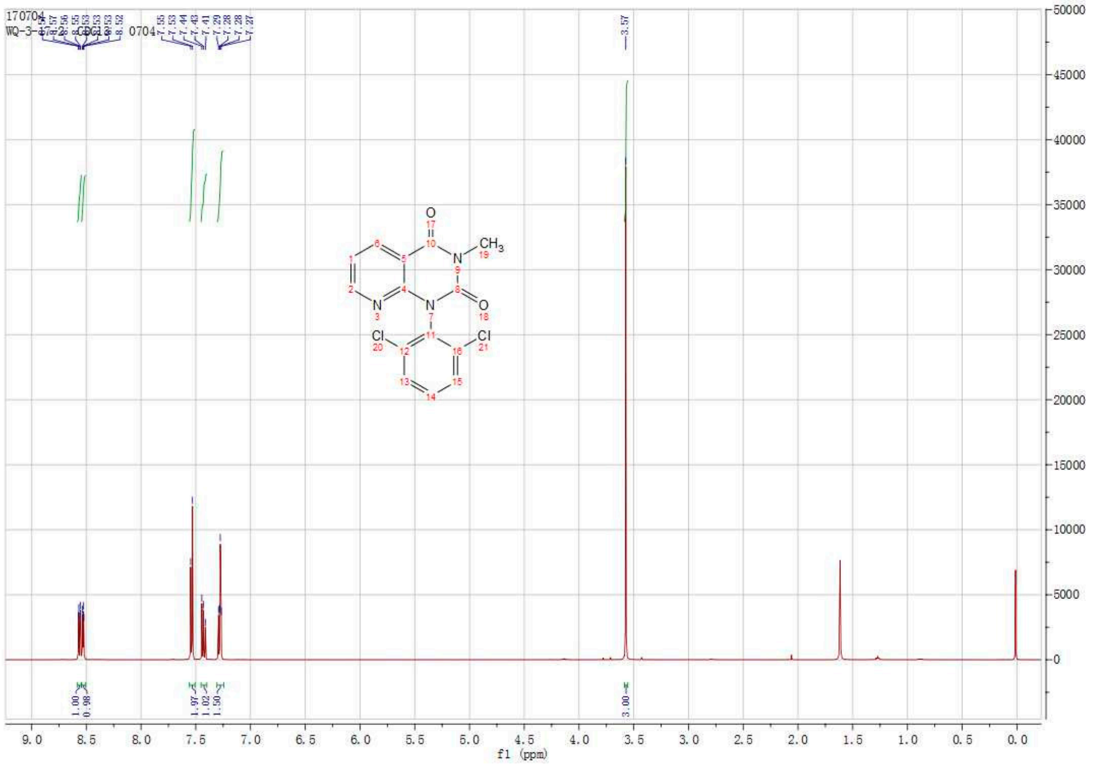

The ^1H NMR of compound 2b

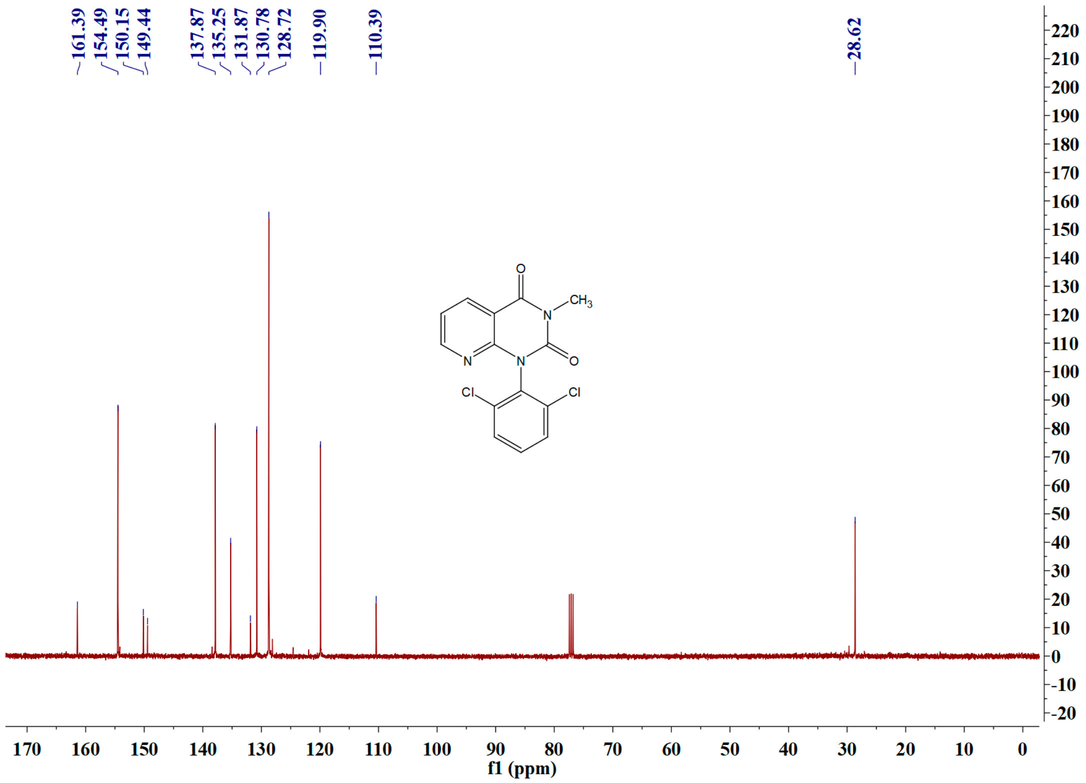

The ^{13}C NMR of compound 2b

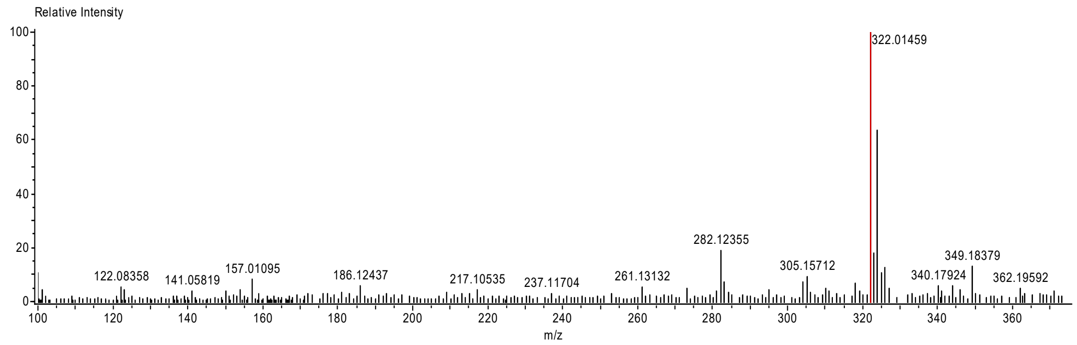

The HRMS of compound 2b

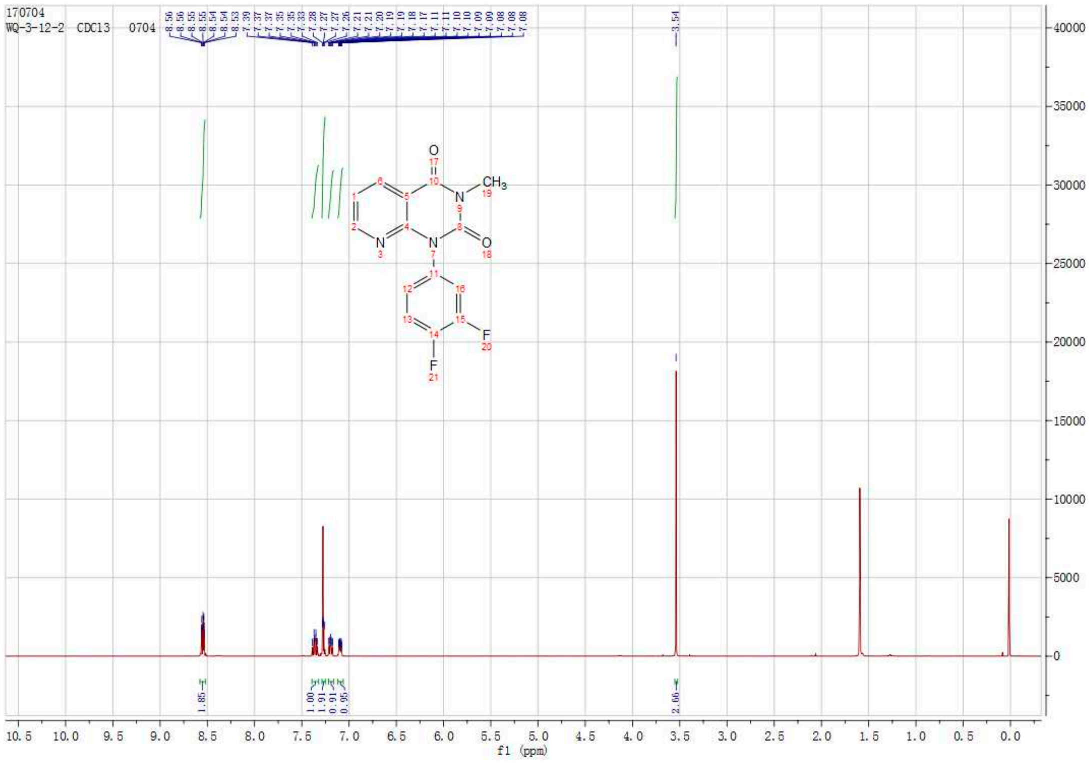

The ^{1}H NMR of compound 2c

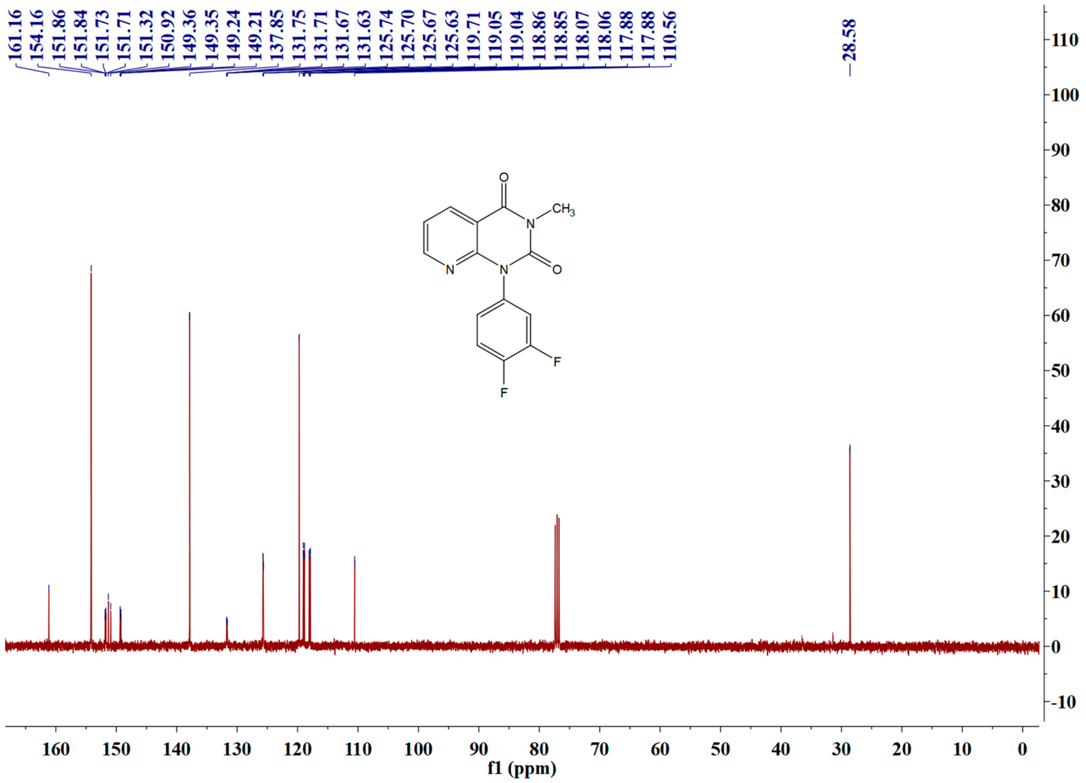

The ^{13}C NMR of compound 2c

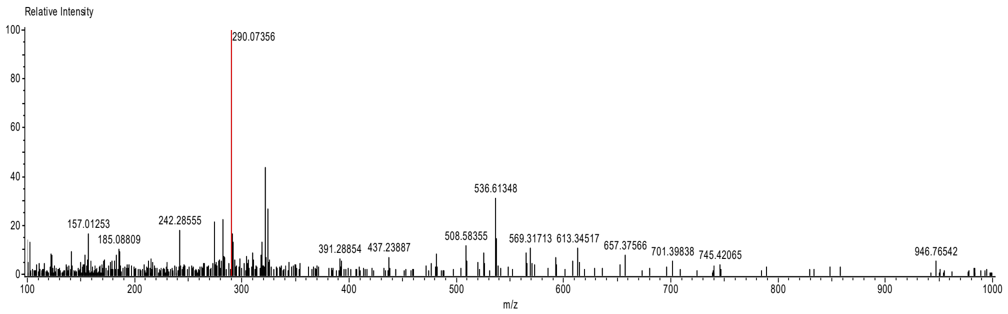

The HRMS of compound 2C

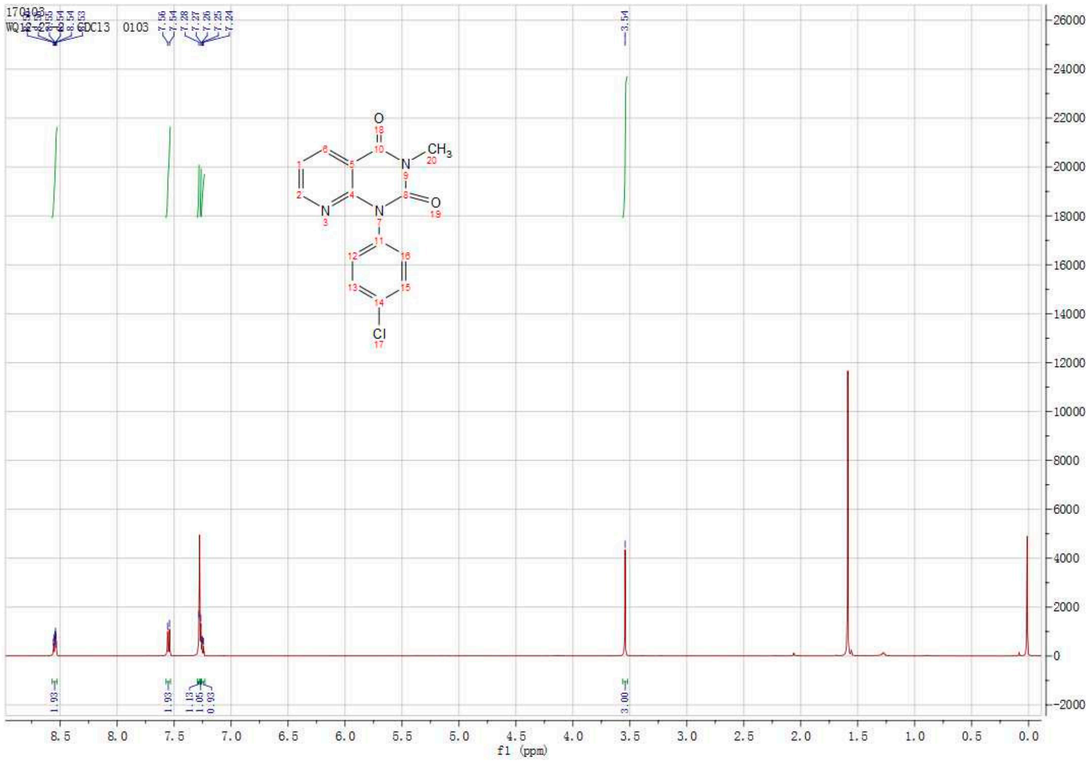

The ^{1}H NMR of compound 2d

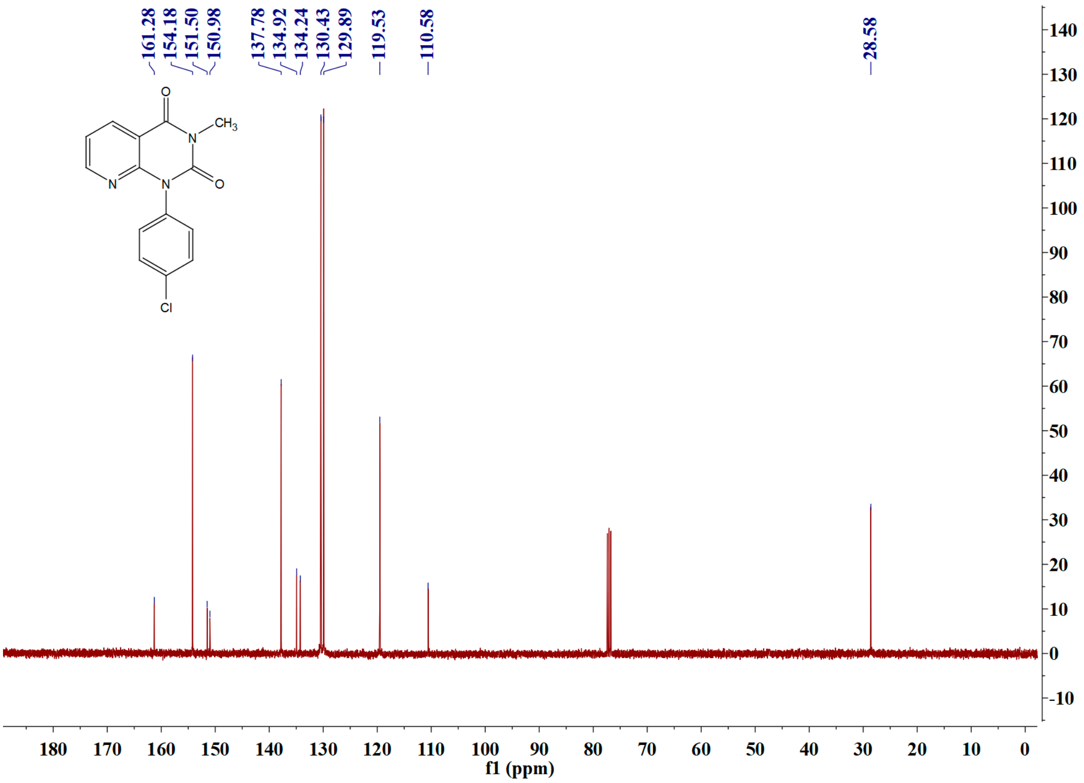

The ^{13}C NMR of compound 2d

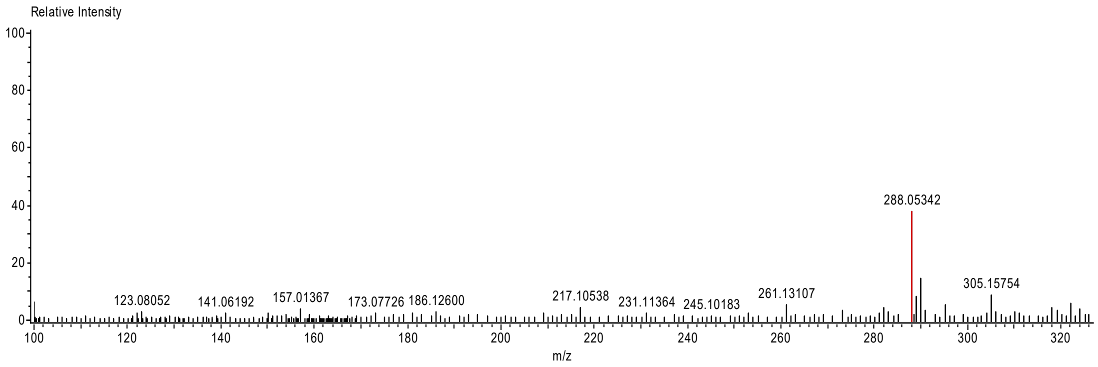

The HRMS of compound 2d

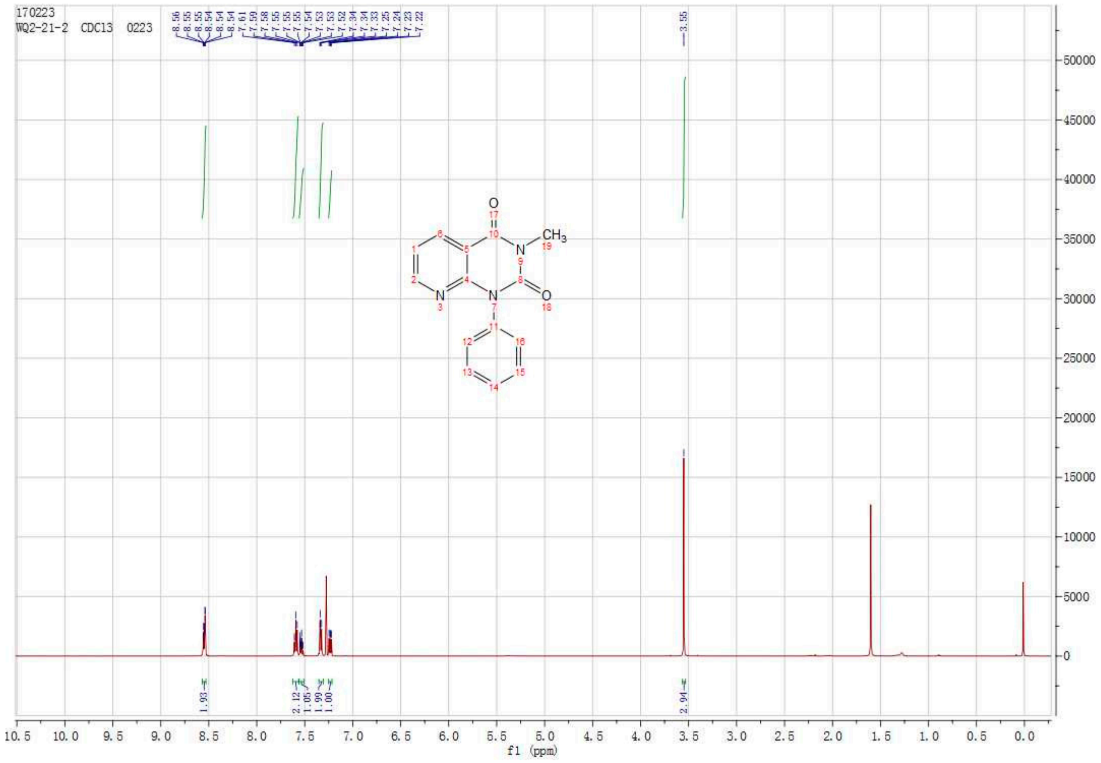

The ^{1}H NMR of compound 2e

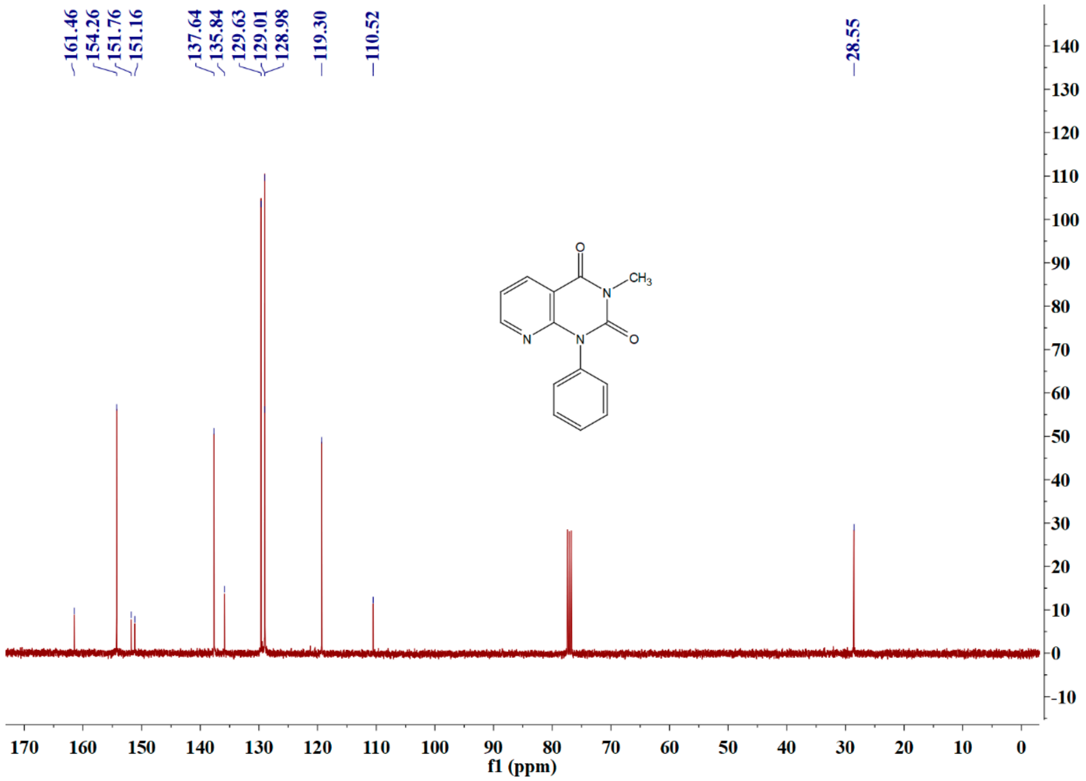

The ^{13}C NMR of compound 2e

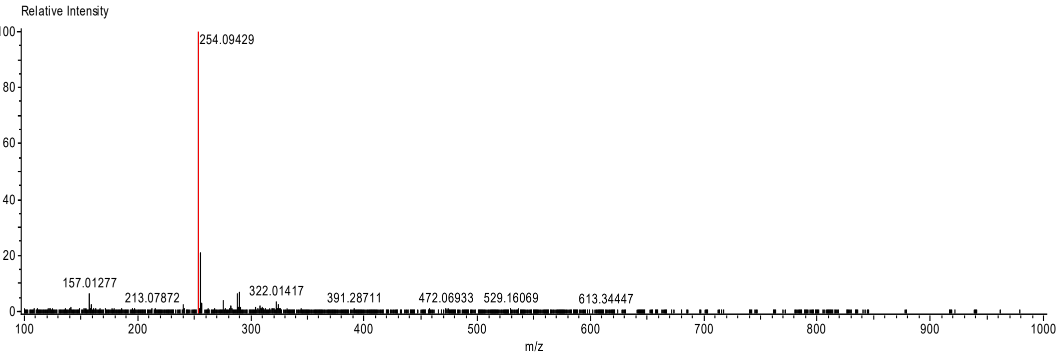

The HRMS of compound 2e

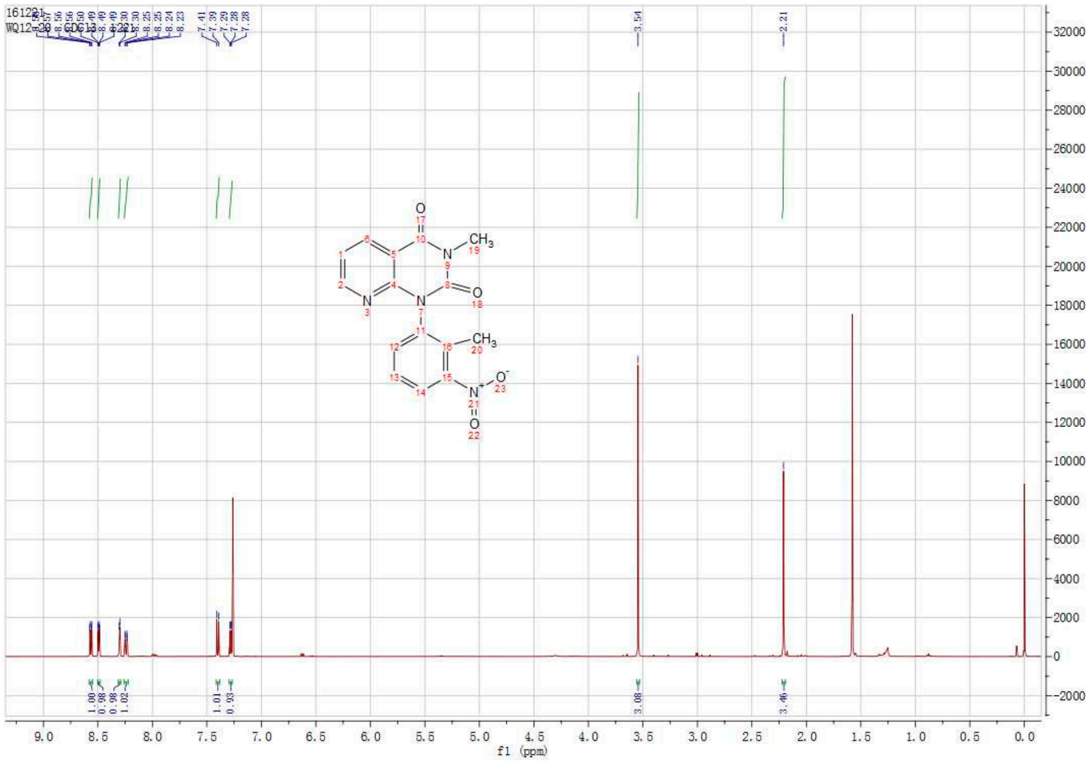

The ^{1}H NMR of compound 2f

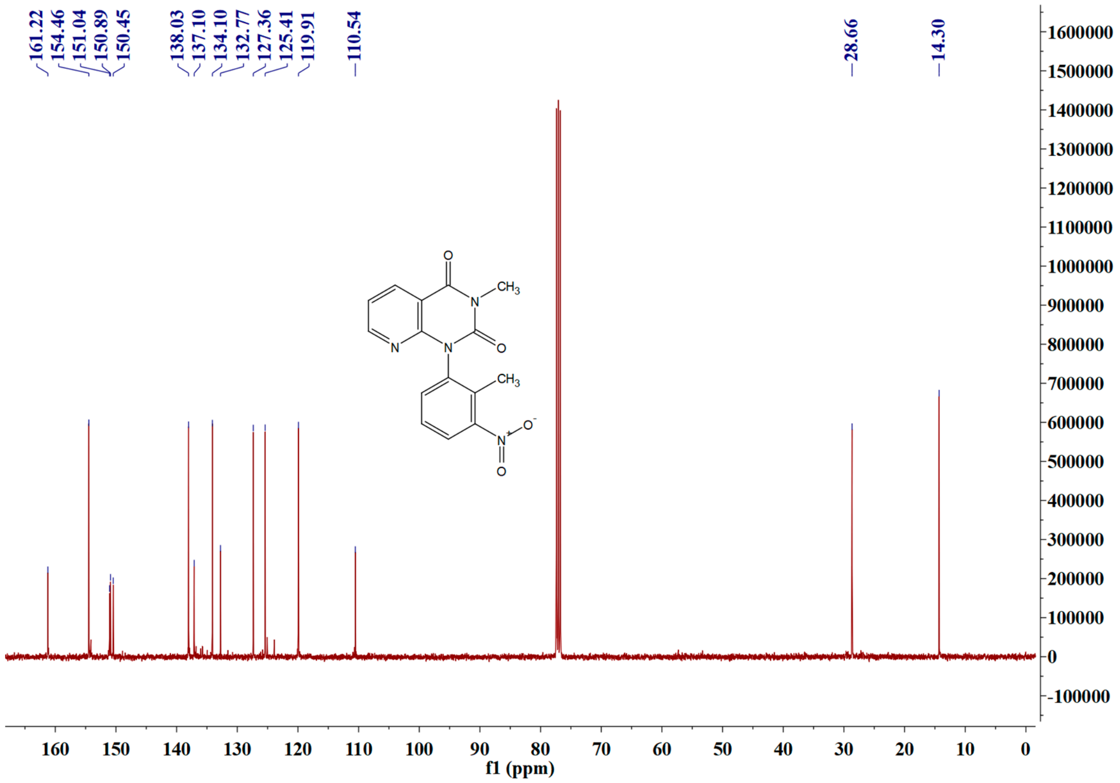

The ^{13}C NMR of compound 2f

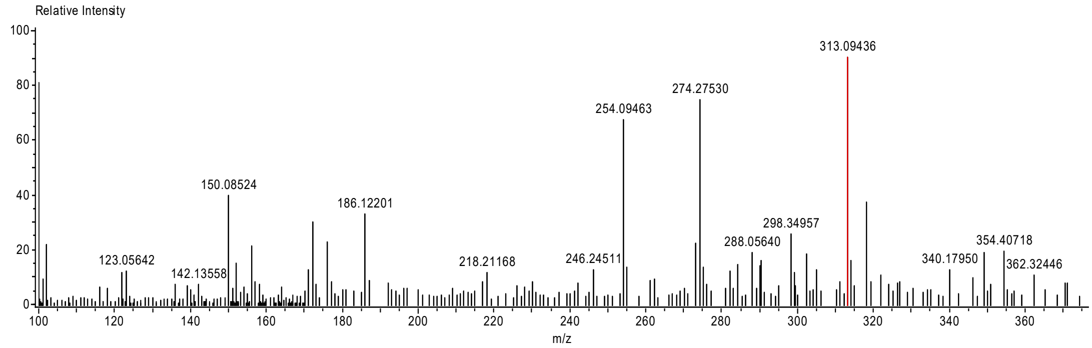

The HRMS of compound 2F

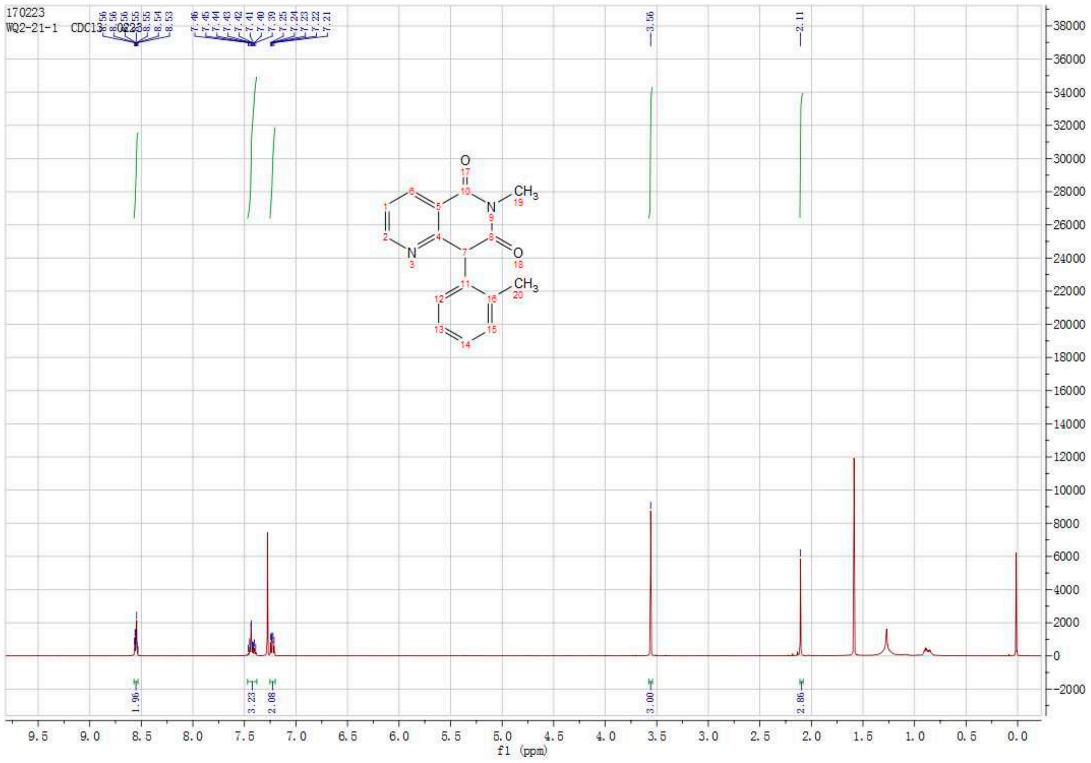

The ^{1}H NMR of compound 2g

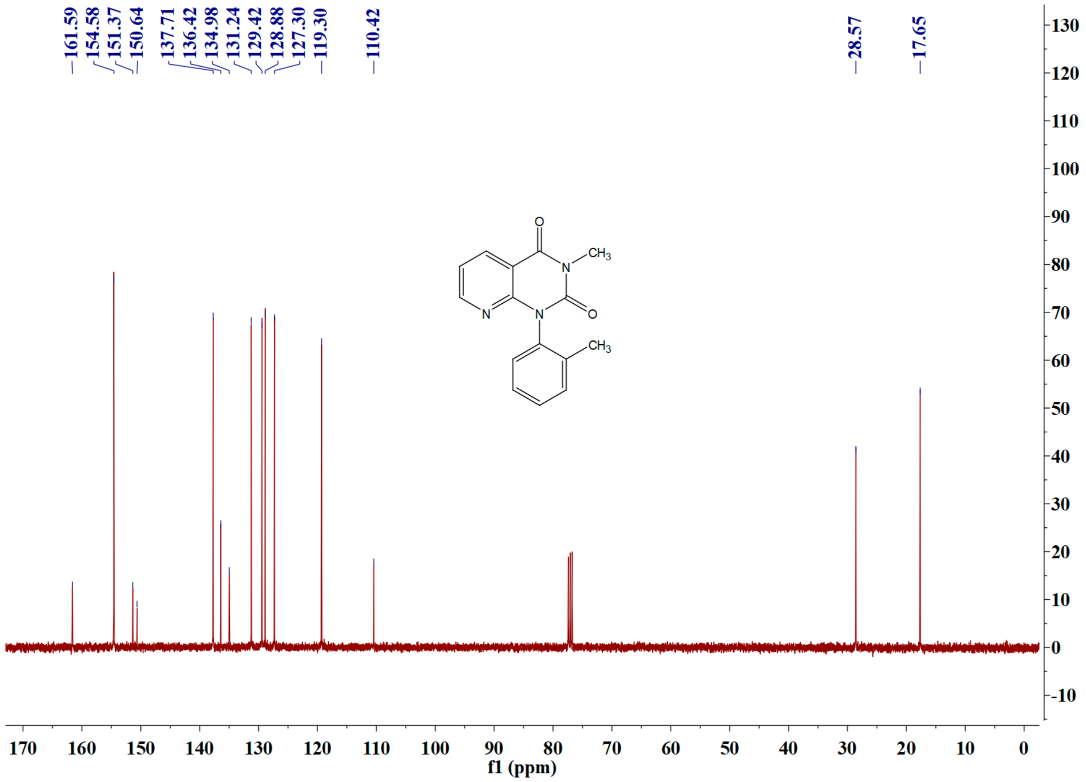

The ^{13}C NMR of compound 2g

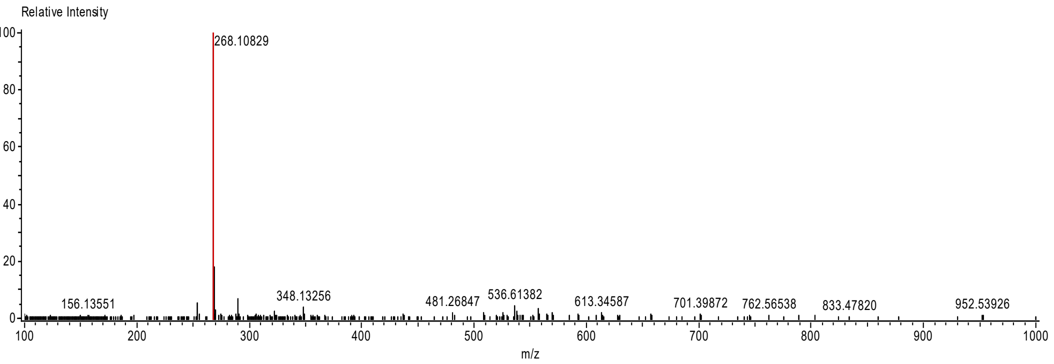

The HRMS of compound 2g

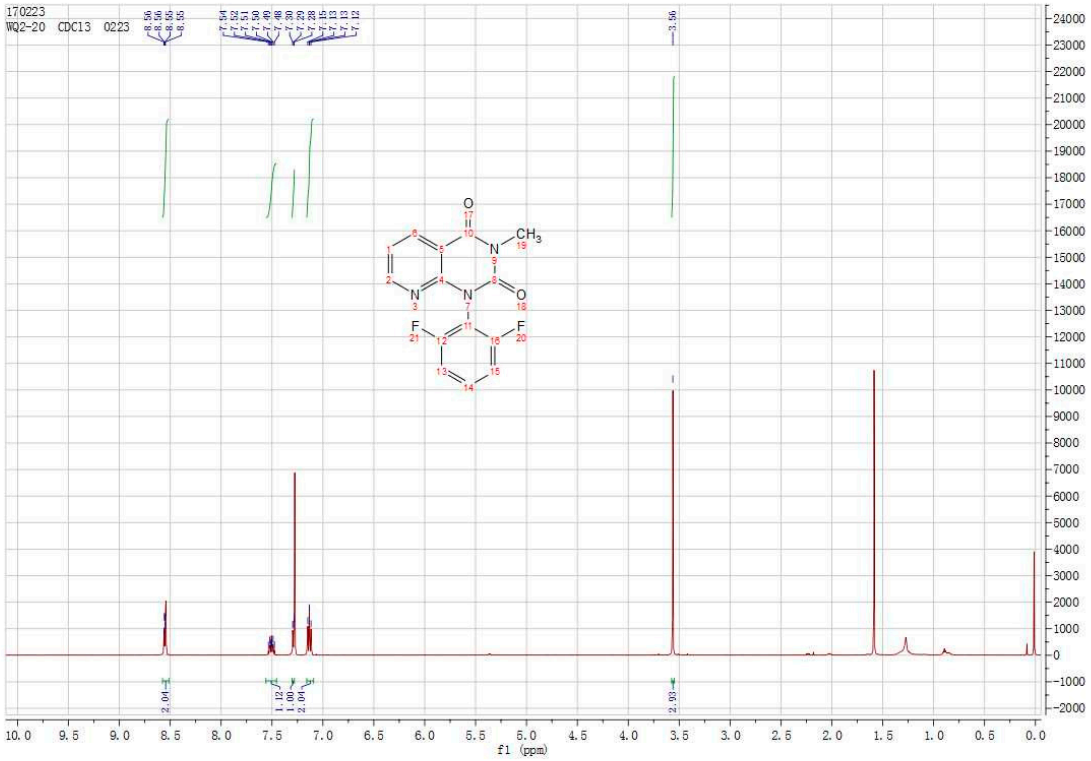

The ^{1}H NMR of compound 2h

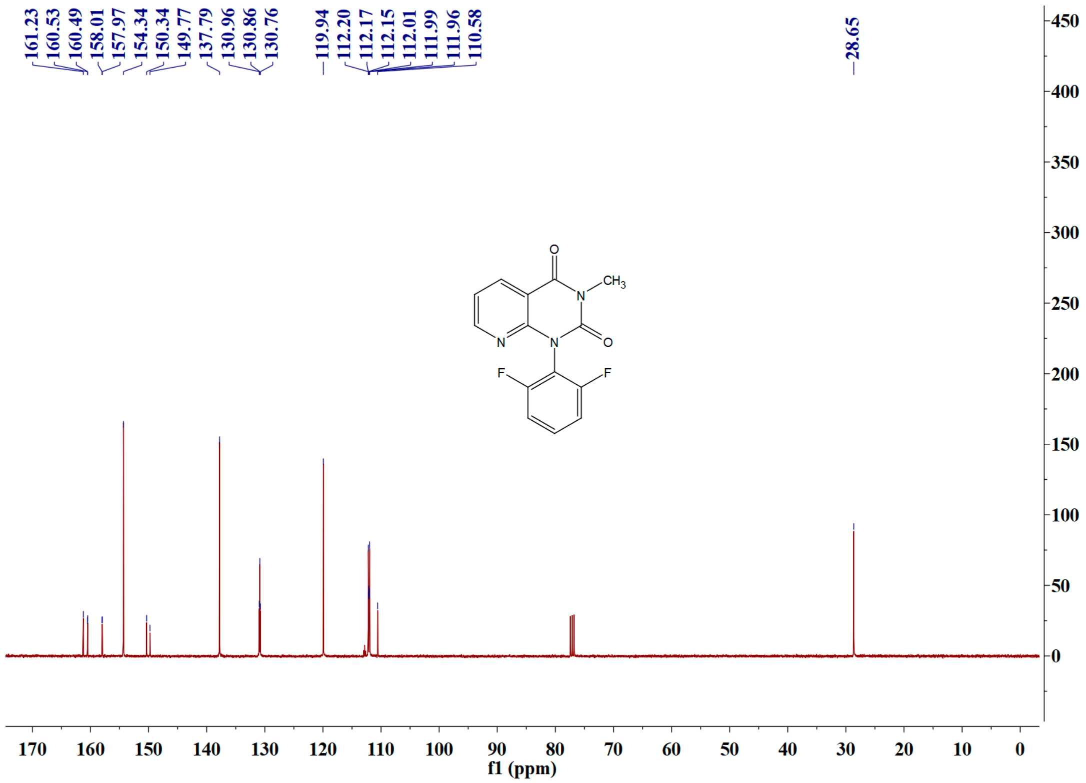

The ^{13}C NMR of compound 2h

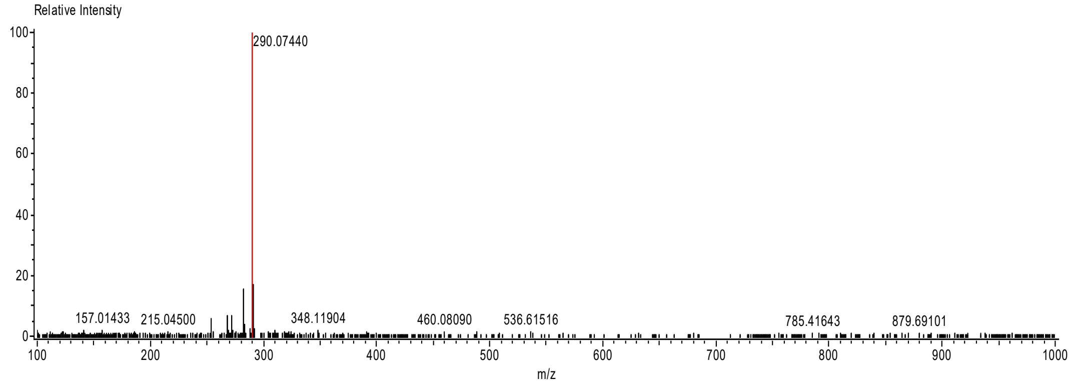

The HRMS of compound 2h

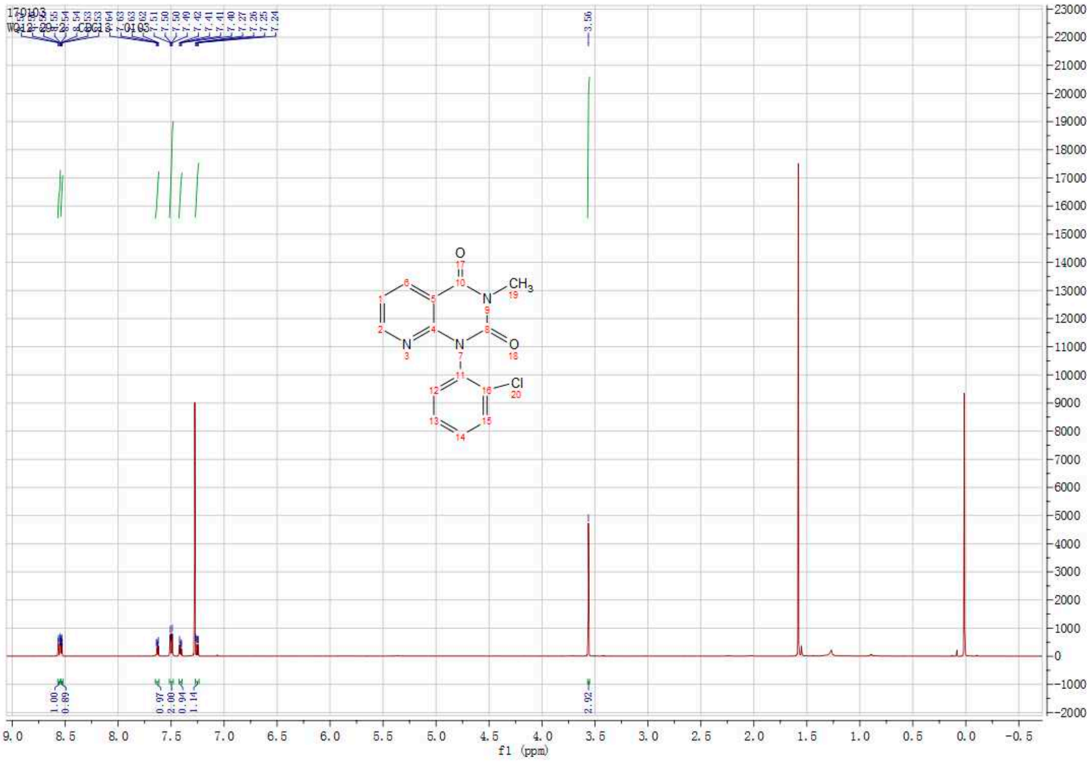

Figure 15 The ^{1}H NMR of compound 2i

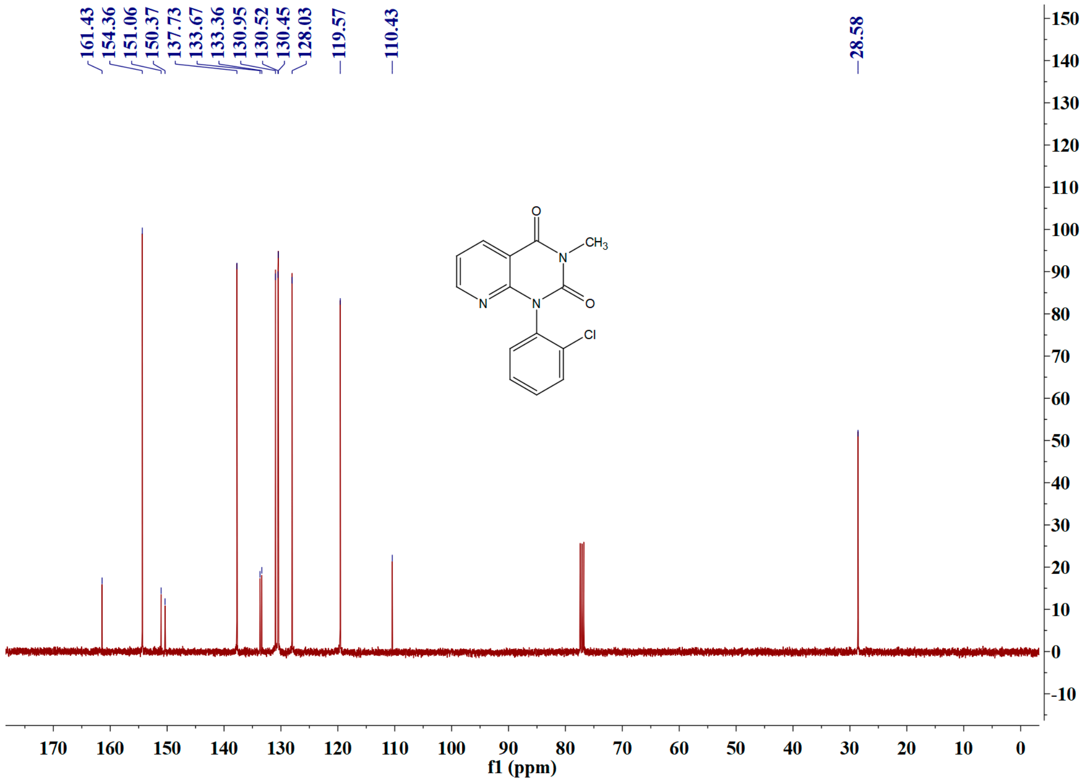

The ^{13}C NMR of compound 2i

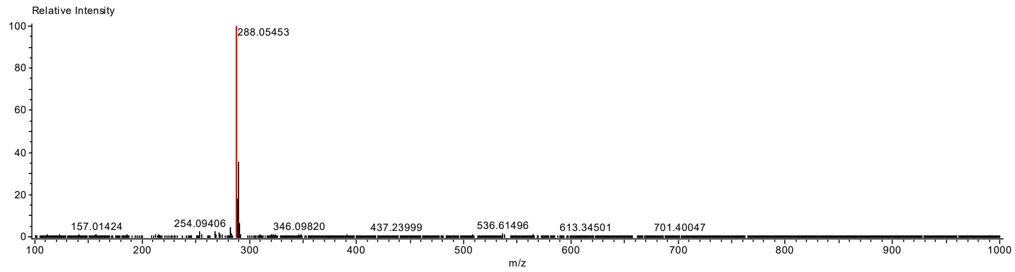

The HRMS of compound 2i

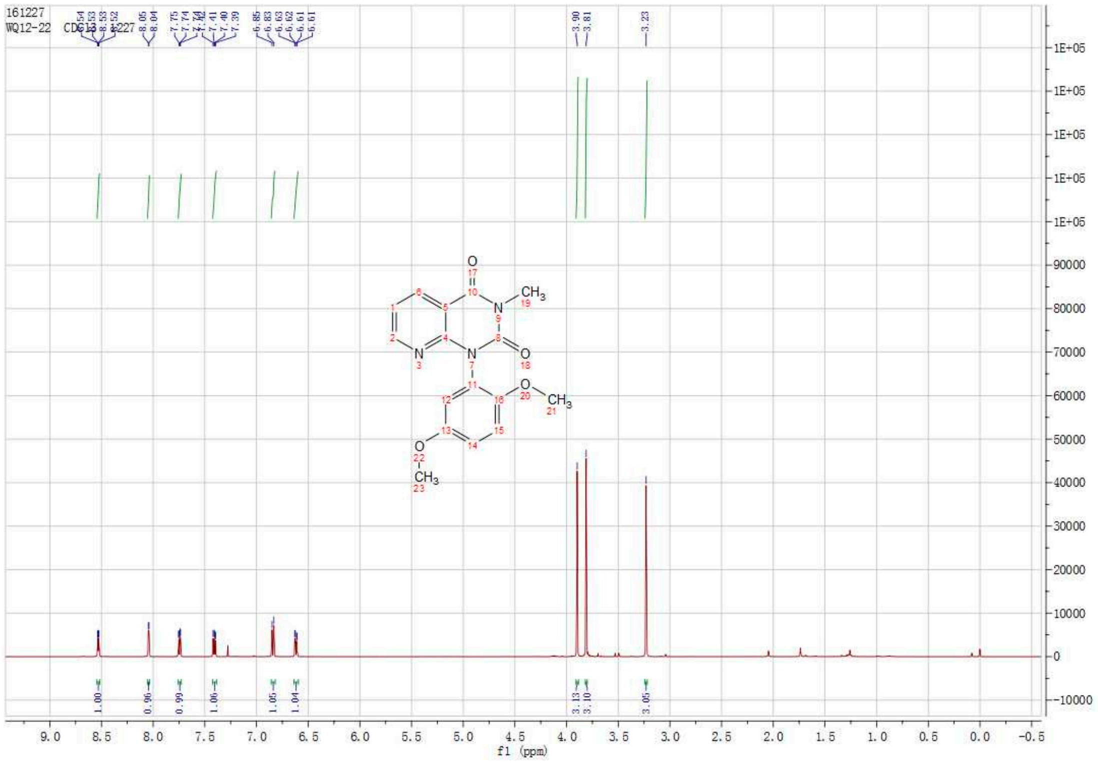

The ¹H NMR of compound 2j

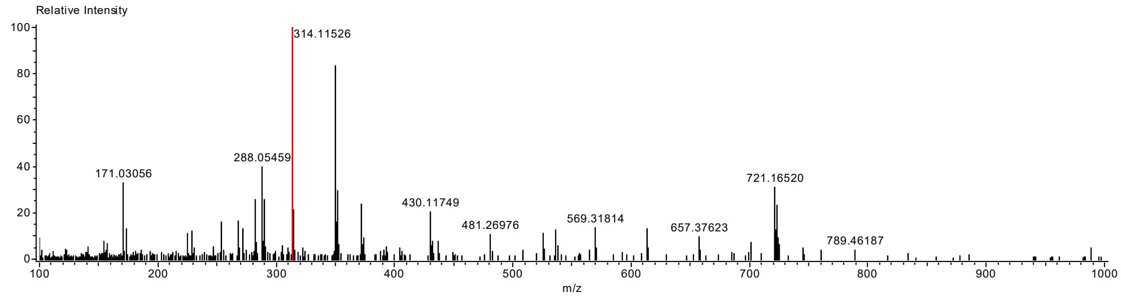

The HRMS of compound 2j

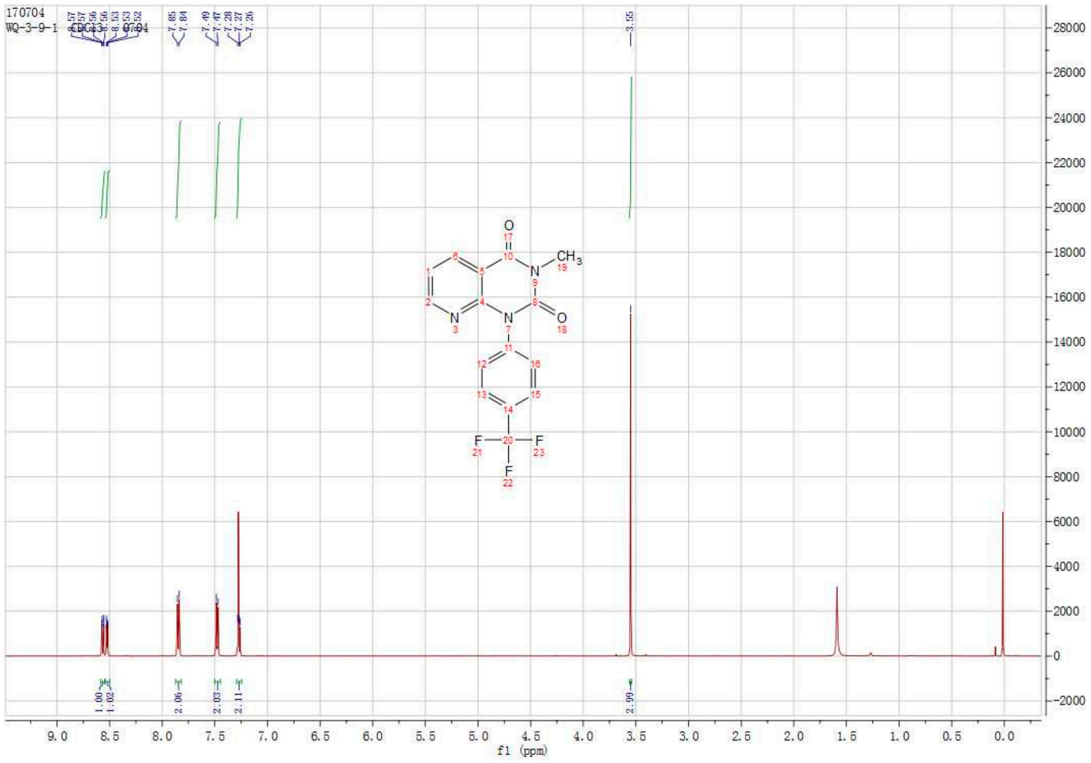

The ^{1}H NMR of compound 2k

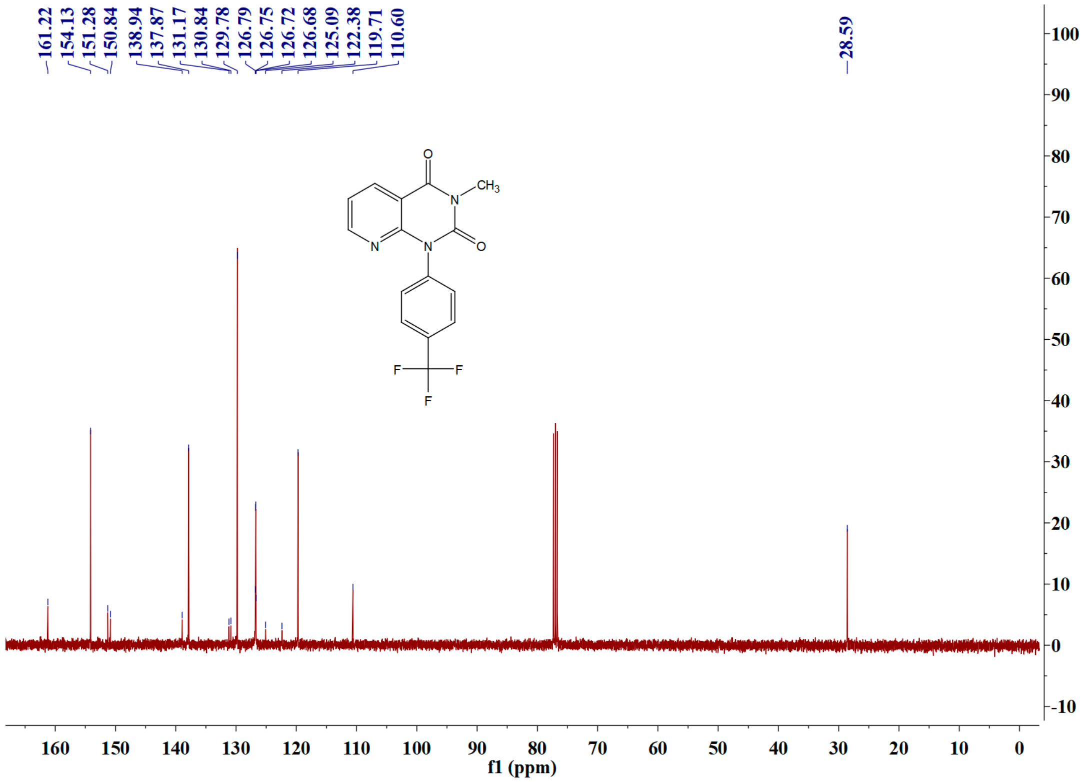

The ^{13}C NMR of compound 2k

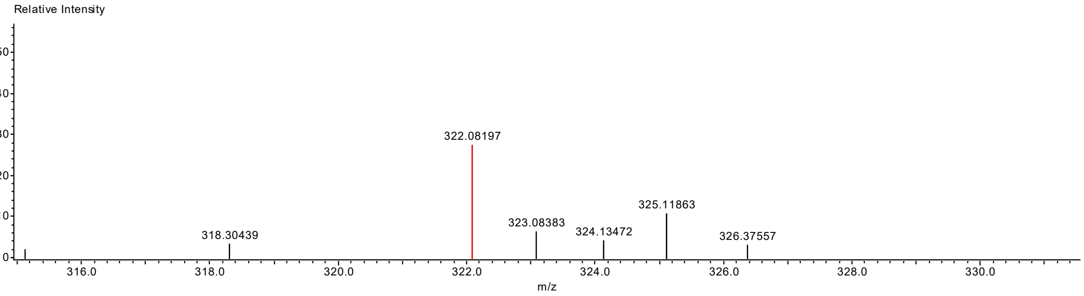

The HRMS of compound 2k

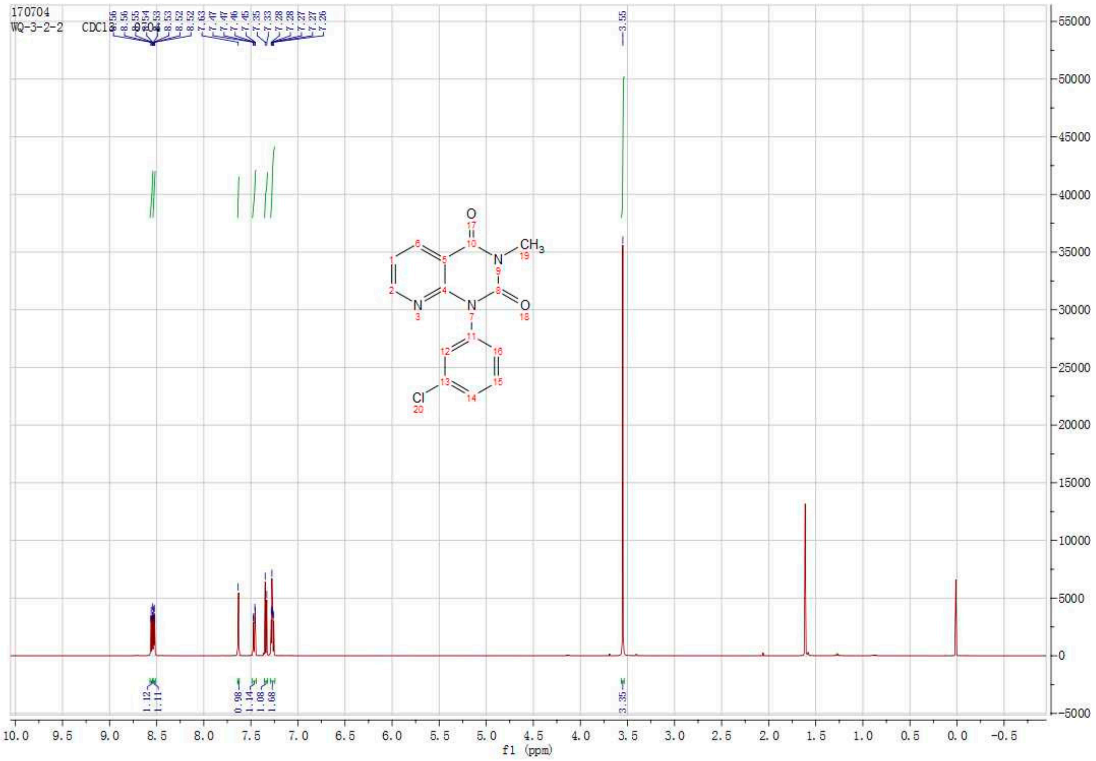

The ^{1}H NMR of compound 2l

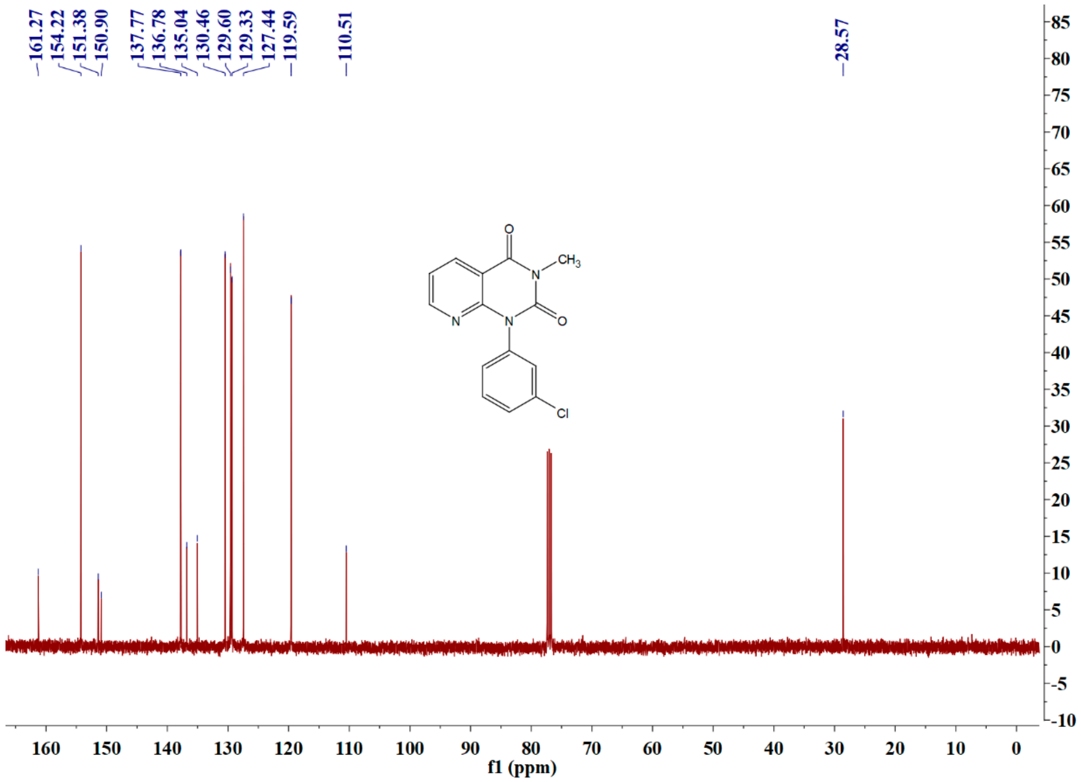

The ^{13}C NMR of compound 2l

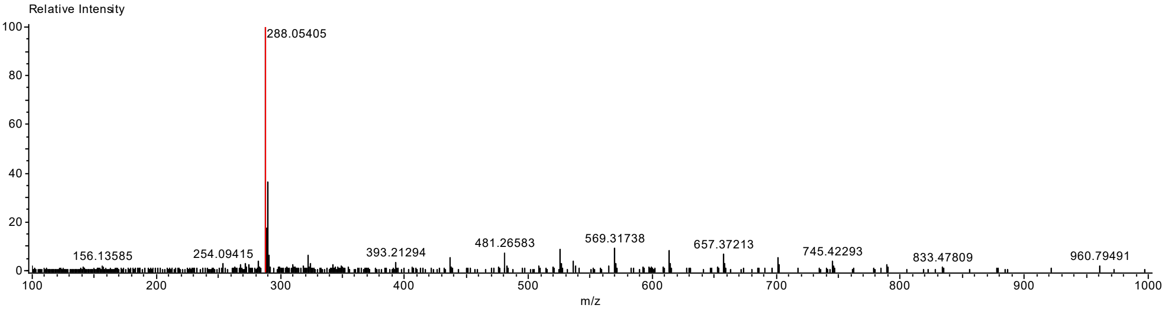

The HRMS of compound 2L

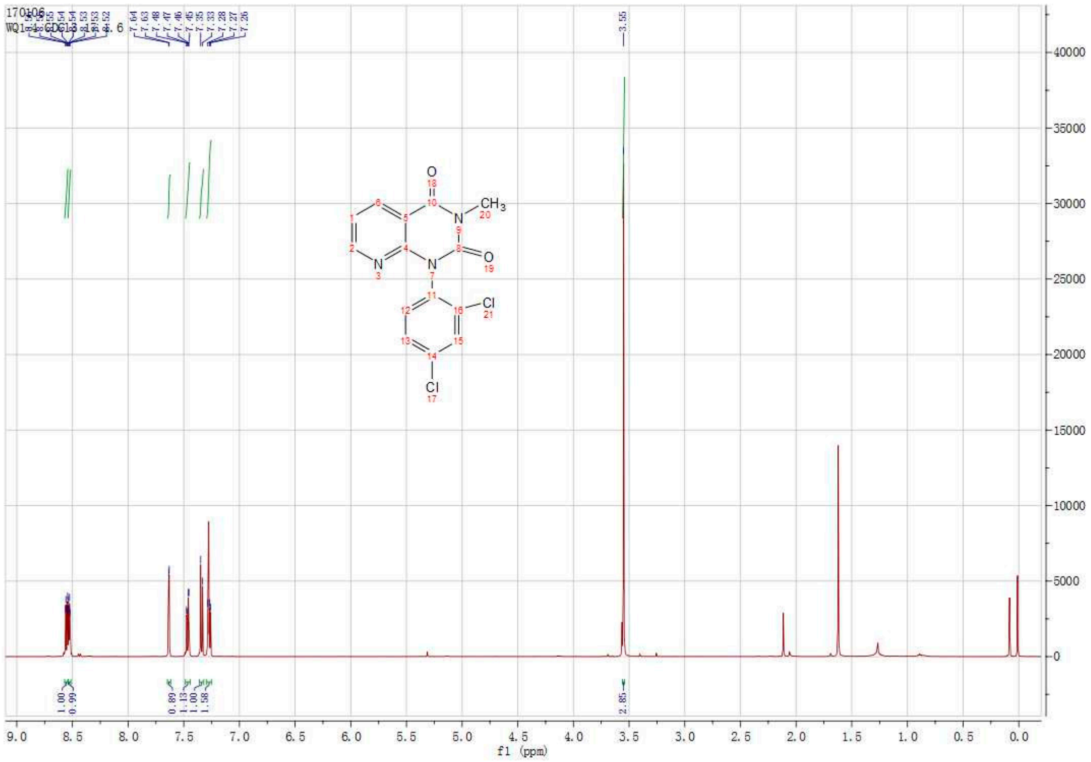

The ^{1}H NMR of compound 2m

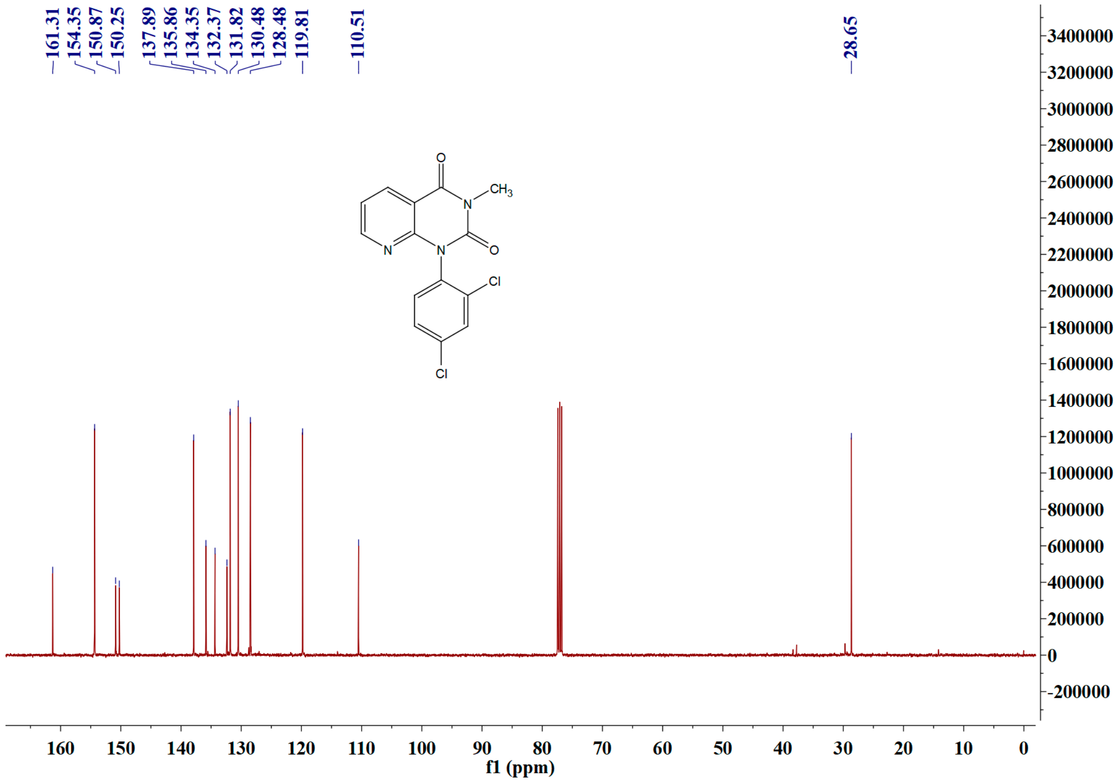

The ^{13}C NMR of compound 2l

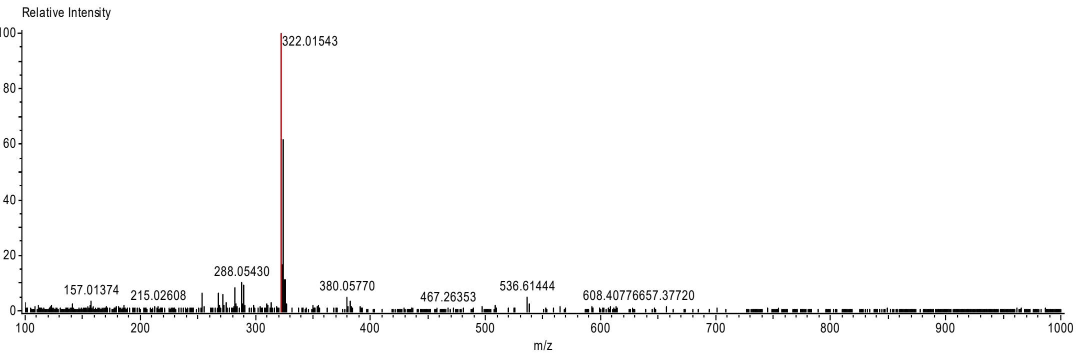

The HRMS of compound 2m

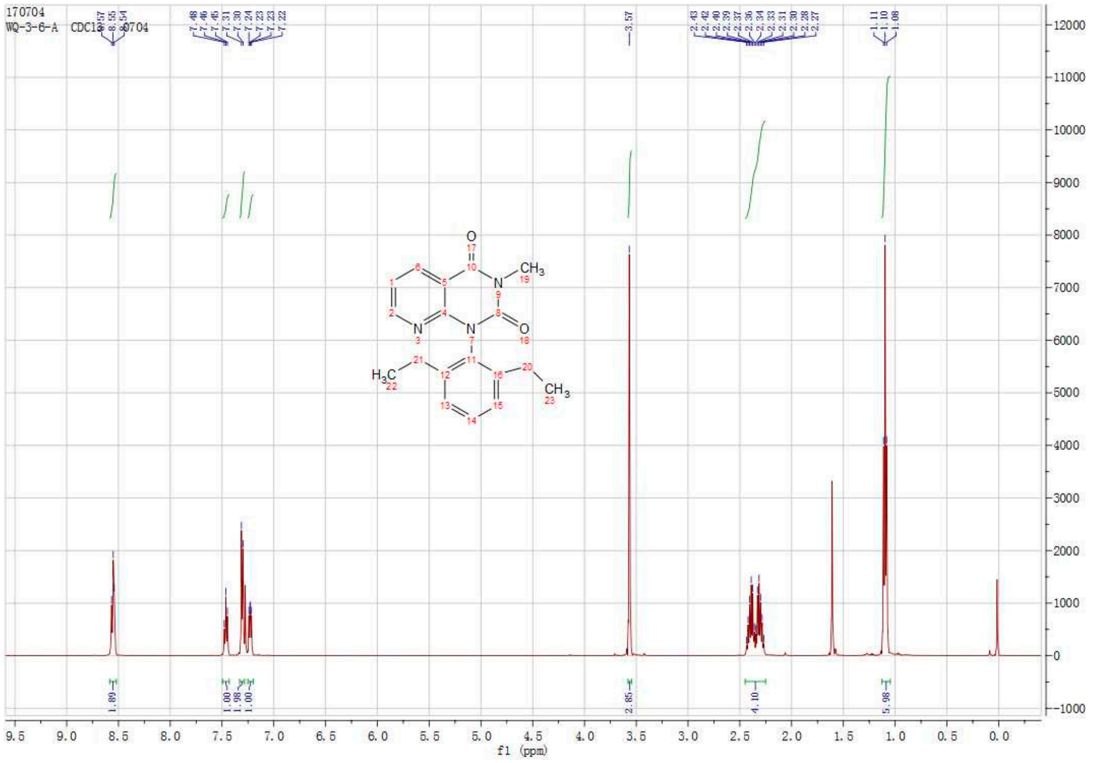

The ^{1}H NMR of compound 2n

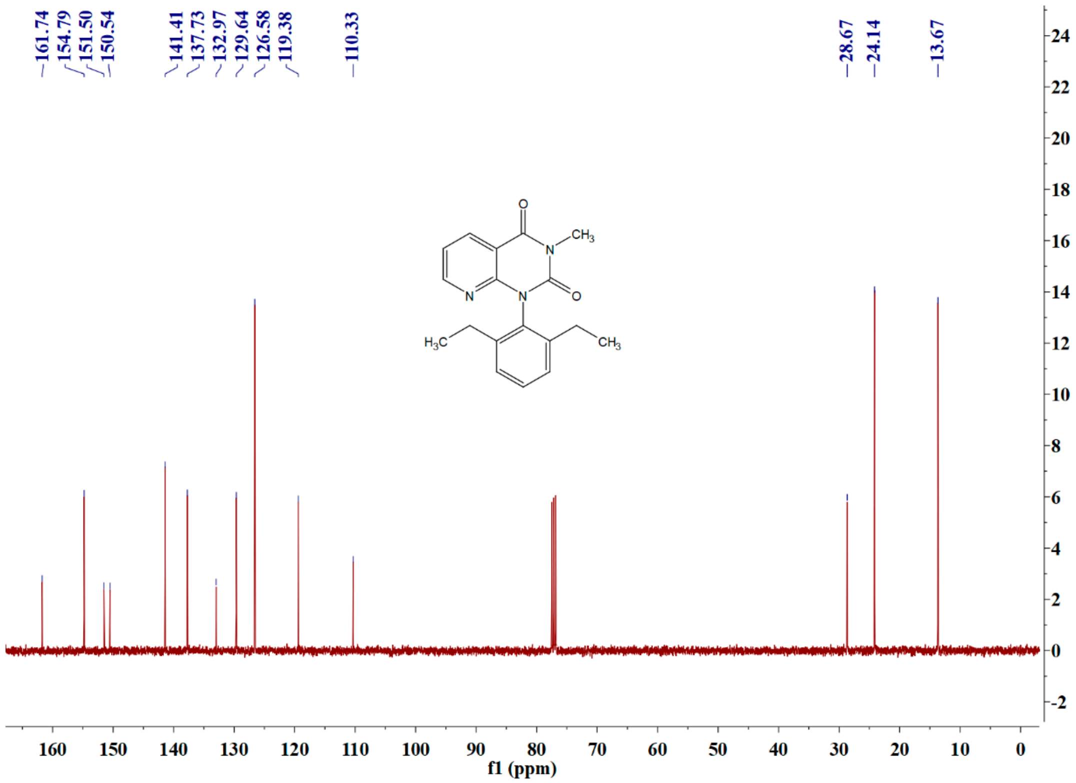

The ^{13}C NMR of compound 2n

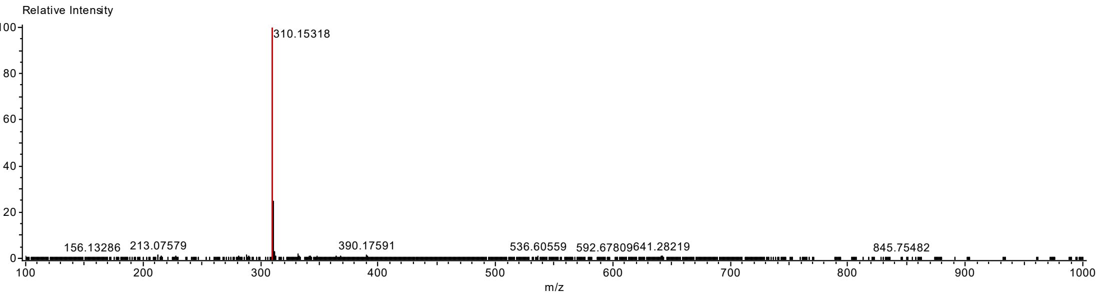

The HRMS of compound 2n

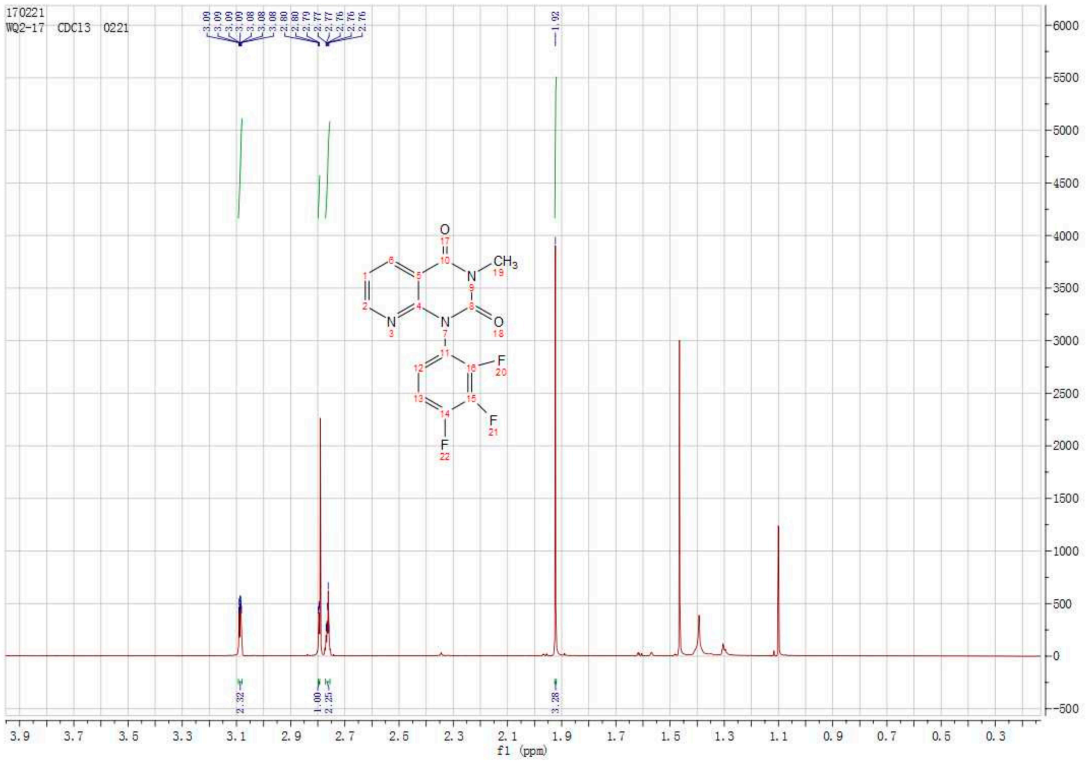

The ¹H NMR of compound 2o

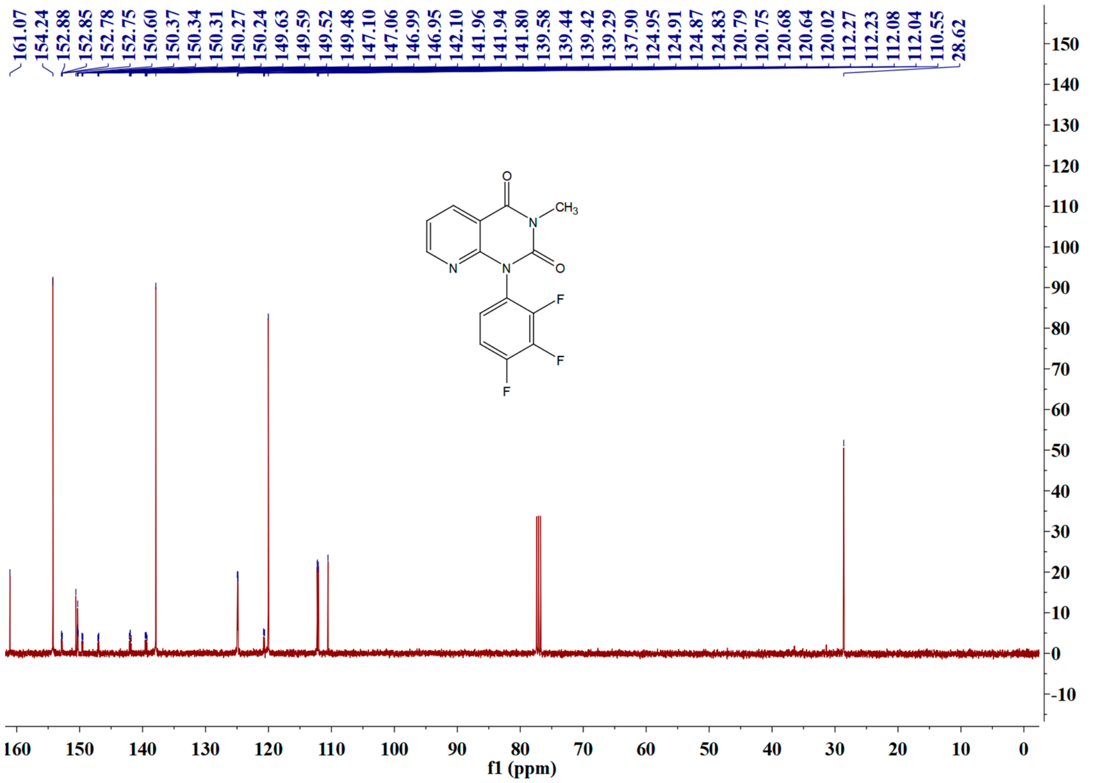

The ¹³C NMR of compound 2o

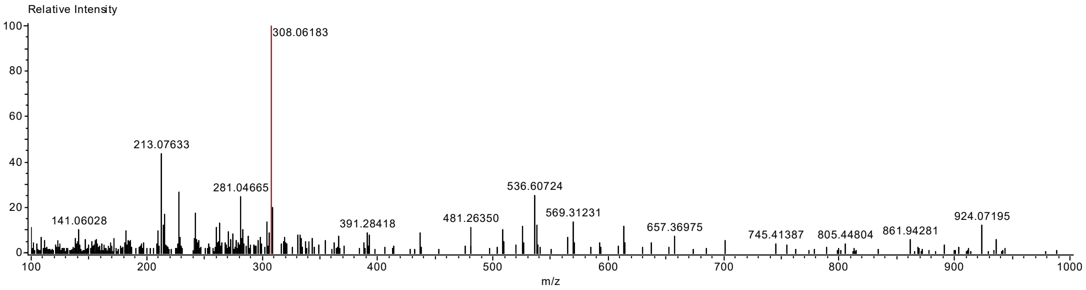

The HRMS of compound 2o

Table S1 Crystal data and structure refinement for 2n.

Identification code	20230421a_0m
Empirical formula	$C_{18}H_{19}N_3O_2$
Formula weight	309.36
Temperature/K	296(2)
Crystal system	monoclinic
Space group	$P2_1/c$
a/Å	8.821(5)
b/Å	12.379(7)
c/Å	15.399(8)
α/°	90
β/°	100.522(18)
γ/°	90
Volume/Å^3	1653.2(16)
Z	4
ρ_{calc}g/cm^3	1.243
μ/mm^{-1}	0.083
F(000)	656.0
Crystal size/mm^3	0.52 × 0.46 × 0.42
Radiation	MoKα (λ = 0.71073)
2Θ range for data collection/°	5.382 to 60.004
Index ranges	-12 ≤ h ≤ 12, -17 ≤ k ≤ 17, -21 ≤ l ≤ 21
Reflections collected	40577
Independent reflections	4763 [R_{int} = 0.0531, R_{sigma} = 0.0358]
Data/restraints/parameters	4763/0/232
Goodness-of-fit on F^2	1.030
Final R indexes [I>=2σ (I)]	R_1 = 0.0599, wR_2 = 0.1531
Final R indexes [all data]	R_1 = 0.1025, wR_2 = 0.1767
Largest diff. peak/hole / e Å^{-3}	0.27/-0.25

Table S2 Fractional Atomic Coordinates (×10^4) and Equivalent Isotropic Displacement Parameters (Å^2×10^3) for 2n. U_{eq} is defined as 1/3 of of the trace of the orthogonalised U_{IJ} tensor.

Atom	*x*	*y*	*z*	U(eq)
O1	7024.0(13)	9024.3(12)	815.4(8)	67.1(4)
O2	2085.6(12)	8060.3(12)	-335.9(8)	62.7(4)
N1	4559.7(13)	8541.9(11)	245.7(8)	40.2(3)
N2	3948.3(12)	7590.5(11)	-1107.2(8)	41.0(3)
N3	5848.7(14)	7064.6(11)	-1889.3(9)	47.6(3)
C1	5505.2(14)	7566.5(12)	-1183.3(9)	36.3(3)
C2	6602.9(15)	8061.3(12)	-534.1(9)	38.6(3)
C3	8141.3(16)	8033.4(15)	-640.8(11)	49.2(4)
C4	8515.8(17)	7525.3(15)	-1365.2(12)	54.9(5)
C5	7340.9(18)	7057.1(15)	-1965.5(12)	53.5(4)
C6	3435.0(16)	8065.4(13)	-398.2(10)	41.8(4)
C7	4027.3(19)	9044.3(15)	1002.8(10)	51.0(4)
C8	6128.3(16)	8578.3(13)	228.8(10)	42.6(4)
C9	2787.7(15)	7086.2(14)	-1784.2(9)	41.2(4)
C10	2449.2(16)	5994.9(15)	-1691.0(11)	49.4(4)
C11	1347.3(19)	5529.5(17)	-2355.0(12)	60.1(5)
C12	607.9(18)	6130.8(19)	-3054.9(12)	63.4(5)
C13	949.2(18)	7207.5(18)	-3119.5(11)	58.3(5)
C14	2066.3(16)	7718.2(15)	-2487.2(10)	47.7(4)
C15	2475(2)	8894.5(16)	-2594.3(14)	65.1(5)
C16	3796(5)	9086(4)	-3043(3)	72.9(14)
C16A	2860(30)	9110(13)	-3491(10)	248(13)
C17	3190(2)	5347.6(19)	-890.1(15)	75.0(6)
C18	2642(12)	4333(6)	-761(7)	187(6)
C18A	1951(9)	5039(9)	-293(5)	84(3)

Table S3 Anisotropic Displacement Parameters (Å^2×10^3) for 2n. The Anisotropic displacement factor exponent takes the form: $-2\pi^2[h^2a^{*2}U_{11}+2hka^*b^*U_{12}+\ldots]$.

Atom	U_{11}	U_{22}	U_{33}	U_{23}	U_{13}	U_{12}
O1	43.3(6)	105.6(11)	48.4(7)	-23.0(7)	-1.9(5)	-14.8(6)
O2	29.4(5)	105.3(11)	55.8(7)	-22.1(7)	14.3(5)	-8.1(6)
N1	32.6(6)	55.0(8)	33.0(6)	-3.4(5)	6.0(5)	-3.5(5)
N2	21.8(5)	62.2(8)	38.3(6)	-9.7(6)	4.0(4)	-4.3(5)
N3	29.8(6)	62.2(8)	51.4(8)	-13.7(6)	9.3(5)	-0.9(6)
C1	23.5(6)	46.1(8)	39.0(7)	-0.3(6)	4.7(5)	-0.6(5)
C2	25.8(6)	49.6(8)	38.8(7)	1.3(6)	2.1(5)	-1.9(6)

C3	25.6(7)	68.9(11)	51.2(9)	-1.5(8)	1.7(6)	-6.1(7)
C4	25.8(7)	77.9(12)	63.1(11)	-4.1(9)	13.4(7)	0.5(7)
C5	34.3(8)	71.0(11)	58.2(10)	-12.6(8)	16.3(7)	3.2(7)
C6	29.1(7)	59.1(9)	37.5(7)	-4.1(7)	6.4(5)	-2.3(6)
C7	49.9(9)	66.9(11)	38.2(8)	-6.7(7)	13.9(7)	-3.0(8)
C8	31.4(7)	58.2(9)	36.2(7)	0.4(7)	0.8(5)	-4.2(6)
C9	20.5(6)	64.5(10)	38.3(7)	-9.2(7)	4.4(5)	-3.4(6)
C10	31.4(7)	70.7(11)	45.1(8)	-4.0(8)	4.8(6)	-9.5(7)
C11	39.5(8)	81.5(13)	58.7(11)	-11.3(9)	7.2(7)	-22.6(8)
C12	31.7(8)	110.1(16)	45.8(9)	-21.4(10)	0.5(7)	-15.1(9)
C13	34.9(8)	98.5(15)	38.9(8)	-4.2(9)	-0.1(6)	9.4(9)
C14	29.5(7)	73.7(11)	40.3(8)	-6.2(7)	7.6(6)	7.2(7)
C15	62.8(11)	69.3(12)	61.3(11)	0.5(9)	6.6(9)	18.1(9)
C16	83(3)	57(2)	85(3)	16(2)	32(2)	10.2(18)
C16A	510(40)	143(11)	127(12)	-29(9)	163(18)	-151(18)
C17	67.3(12)	83.6(15)	67.9(13)	11.1(11)	-4.0(10)	-7.1(11)
C18	209(9)	111(5)	190(9)	85(6)	-102(8)	-86(6)
C18A	96(5)	93(6)	66(4)	15(4)	21(3)	-20(4)

Table S4 Bond Lengths for 2n.

Atom	Atom	Length/Å	Atom	Atom	Length/Å
O1	C8	1.2183(18)	C4	C5	1.384(2)
O2	C6	1.2115(18)	C9	C10	1.397(3)
N1	C6	1.3987(19)	C9	C14	1.392(2)
N1	C7	1.472(2)	C10	C11	1.400(2)
N1	C8	1.3894(19)	C10	C17	1.515(3)
N2	C1	1.3997(18)	C11	C12	1.373(3)
N2	C6	1.387(2)	C12	C13	1.374(3)
N2	C9	1.4612(18)	C13	C14	1.403(2)
N3	C1	1.334(2)	C14	C15	1.516(3)
N3	C5	1.343(2)	C15	C16	1.479(5)
C1	C2	1.400(2)	C15	C16A	1.503(12)
C2	C3	1.397(2)	C17	C18	1.373(5)
C2	C8	1.464(2)	C17	C18A	1.598(6)
C3	C4	1.373(2)			

Table S5 Bond Angles for 2n.

Atom	Atom	Atom	Angle/°	Atom	Atom	Atom	Angle/°
C6	N1	C7	116.88(12)	O1	C8	N1	120.93(14)
C8	N1	C6	125.47(12)	O1	C8	C2	123.44(14)

C8	N1	C7	117.65(12)	N1	C8	C2	115.62(12)
C1	N2	C9	120.09(12)	C10	C9	N2	118.36(13)
C6	N2	C1	122.77(12)	C14	C9	N2	118.43(15)
C6	N2	C9	117.12(11)	C14	C9	C10	123.21(14)
C1	N3	C5	116.52(13)	C9	C10	C11	117.13(16)
N3	C1	N2	116.77(12)	C9	C10	C17	121.78(15)
N3	C1	C2	123.74(13)	C11	C10	C17	121.04(18)
C2	C1	N2	119.49(13)	C12	C11	C10	121.15(19)
C1	C2	C8	120.16(13)	C11	C12	C13	120.26(15)
C3	C2	C1	117.74(14)	C12	C13	C14	121.48(17)
C3	C2	C8	122.10(13)	C9	C14	C13	116.74(17)
C4	C3	C2	119.35(14)	C9	C14	C15	122.71(15)
C3	C4	C5	118.22(14)	C13	C14	C15	120.53(16)
N3	C5	C4	124.43(16)	C16	C15	C14	115.3(2)
O2	C6	N1	121.73(14)	C16A	C15	C14	111.6(5)
O2	C6	N2	121.80(13)	C10	C17	C18A	110.9(3)
N2	C6	N1	116.47(12)	C18	C17	C10	119.4(3)

Table S6 Torsion Angles for 2n.

A	B	C	D	Angle/°	A	B	C	D	Angle/°
N2	C1	C2	C3	-179.07(14)	C7	N1	C6	N2	-179.75(14)
N2	C1	C2	C8	1.4(2)	C7	N1	C8	O1	-1.0(2)
N2	C9	C10	C11	-179.21(14)	C7	N1	C8	C2	179.58(14)
N2	C9	C10	C17	3.2(2)	C8	N1	C6	O2	179.61(16)
N2	C9	C14	C13	-179.45(12)	C8	N1	C6	N2	0.2(2)
N2	C9	C14	C15	2.2(2)	C8	C2	C3	C4	179.24(16)
N3	C1	C2	C3	0.6(2)	C9	N2	C1	N3	-0.3(2)
N3	C1	C2	C8	-178.90(15)	C9	N2	C1	C2	179.42(13)
C1	N2	C6	O2	-178.60(15)	C9	N2	C6	O2	0.4(2)
C1	N2	C6	N1	0.8(2)	C9	N2	C6	N1	179.82(13)
C1	N2	C9	C10	88.59(17)	C9	C10	C11	C12	-1.6(2)
C1	N2	C9	C14	-91.83(17)	C9	C10	C17	C18	169.7(8)
C1	N3	C5	C4	0.3(3)	C9	C10	C17	C18A	111.0(4)
C1	C2	C3	C4	-0.3(2)	C9	C14	C15	C16	85.1(3)
C1	C2	C8	O1	-179.85(15)	C9	C14	C15	C16A	126.7(13)
C1	C2	C8	N1	-0.4(2)	C10	C9	C14	C13	0.1(2)
C2	C3	C4	C5	0.0(3)	C10	C9	C14	C15	-178.29(15)
C3	C2	C8	O1	0.6(3)	C10	C11	C12	C13	0.5(3)
C3	C2	C8	N1	-179.96(14)	C11	C10	C17	C18	-7.9(8)
C3	C4	C5	N3	0.0(3)	C11	C10	C17	C18A	-66.6(5)

C5	N3	C1	N2	179.10(14)	C11	C12	C13	C14	0.9(3)
C5	N3	C1	C2	-0.6(2)	C12	C13	C14	C9	-1.2(2)
C6	N1	C8	O1	179.07(16)	C12	C13	C14	C15	177.22(16)
C6	N1	C8	C2	-0.4(2)	C13	C14	C15	C16	-93.2(3)
C6	N2	C1	N3	178.65(14)	C13	C14	C15	C16A	-51.6(13)
C6	N2	C1	C2	-1.6(2)	C14	C9	C10	C11	1.2(2)
C6	N2	C9	C10	-90.43(17)	C14	C9	C10	C17	-176.39(16)
C6	N2	C9	C14	89.15(17)	C17	C10	C11	C12	176.09(18)
C7	N1	C6	O2	-0.3(2)					

Table S7 Hydrogen Atom Coordinates (Å×10^4) and Isotropic Displacement Parameters (Å^2×10^3) for 2n.

Atom	*x*	*y*	*z*	U(eq)
H3	8904	8356	-225	59
H4	9532	7496	-1450	66
H5	7602	6714	-2455	64
H7A	2974	9268	829	76
H7B	4657	9661	1198	76
H7C	4104	8531	1476	76
H11	1112	4800	-2322	72
H12	-127	5808	-3487	76
H13	428	7607	-3593	70
H15A	1578	9259	-2925	78
H15B	2702	9224	-2013	78
H15C	1613	9345	-2512	78
H15D	3352	9086	-2143	78
H16A	4704	8756	-2710	109
H16B	3961	9849	-3087	109
H16C	3580	8777	-3625	109
H16D	3926	8955	-3480	371
H16E	2656	9855	-3643	371
H16F	2232	8659	-3921	371
H17A	4277	5278	-913	90
H17B	3118	5774	-371	90
H17C	4013	5767	-543	90
H17D	3636	4693	-1081	90
H18A	1538	4356	-832	281
H18B	3070	4091	-175	281
H18C	2934	3844	-1185	281
H18D	1599	5684	-46	127

H18E	2418	4568	176	127
H18F	1092	4679	-649	127

Table S8 Atomic Occupancy for 2n.

**Atom**	***Occupancy***	**Atom**	***Occupancy***	**Atom**	***Occupancy***
H15A	0.624(9)	H15B	0.624(9)	H15C	0.376(9)
H15D	0.376(9)	C16	0.624(9)	H16A	0.624(9)
H16B	0.624(9)	H16C	0.624(9)	C16A	0.376(9)
H16D	0.376(9)	H16E	0.376(9)	H16F	0.376(9)
H17A	0.624(9)	H17B	0.624(9)	H17C	0.376(9)
H17D	0.376(9)	C18	0.624(9)	H18A	0.624(9)
H18B	0.624(9)	H18C	0.624(9)	C18A	0.376(9)
H18D	0.376(9)	H18E	0.376(9)	H18F	0.376(9)
